# Development of best practice guidelines for suicide-related crisis response and aftercare in the emergency department or other acute settings: a Delphi expert consensus study

**DOI:** 10.1186/s12888-018-1995-1

**Published:** 2019-01-07

**Authors:** Nicole T. M. Hill, Fiona Shand, Michelle Torok, Lyndal Halliday, Nicola J. Reavley

**Affiliations:** 1 Orygen, the National Centre of Excellence in Youth Mental Health, University of Melbourne, 35 Poplar Rd, Parkville, Victoria 3052 Australia; 2 Black Dog Institute, University of NSW, Hospital Road, Randwick, NSW 2031 Australia; 3 Centre for Mental Health, The Melbourne School of Population and Global Health, University of Melbourne, 207 Bouverie Street, Parkville, Victoria 3010 Australia

**Keywords:** Suicide prevention, Self-harm, Delphi method, Expert consensus, Emergency department, Acute settings

## Abstract

**Background:**

For those who have experienced suicidal behaviour, discharge from the hospital emergency department and other acute settings represents a period of heightened vulnerability for future suicide risk. Current guidelines for suicide response in acute settings often fail to fully address the barriers faced by emergency department personnel who have contact with a person who presents for suicidal behaviour, and have been developed largely without the input of consumers or service users. The aim of the study was to use the Delphi expert consensus method to develop guidelines for staff responding to suicidal presentations in acute settings.

**Methods:**

Systematic searches of academic and grey literature and interviews with key informants were conducted in order to develop a 525-item questionnaire, which comprised actions staff can take when responding to suicide-related presentations in acute settings. This was administered over three rounds to two panels consisting of Australian experts (39 health professionals, 50 consumers with lived experience). Items that reached consensus by at least 80% across both panels were included in the guidelines.

**Results:**

A total of 420 items were rated as essential or important by at least 80% of both panels. The items included strategies that covered initial contact, assessment, referral, discharge and follow-up, staff training, and linkage with community aftercare services. Participation rate across all three rounds was 67.4% (78% consumers, 53.8% professionals).

**Conclusion:**

The guidelines include strategies for responding to suicidal behaviour in acute settings. These guidelines can be used to inform policy development and address barriers to best practice for those working in the area. Future research should investigate ways to optimise implementation of these guidelines in order to improve equal access to quality care for who present to acute settings for suicidal behaviour.

**Electronic supplementary material:**

The online version of this article (10.1186/s12888-018-1995-1) contains supplementary material, which is available to authorized users.

## Background

Suicide causes significant public health burden worldwide. In Australia, suicide is a leading cause of death in Australians aged 15–49 years, accounting for approximately 2800 deaths [1] and over 26,000 hospitalisations annually [2]. However this number significantly underestimates the proportion of people who present to the acute settings (hospital emergency departments and Psychiatric Emergency Care Centres) for suicide-related behaviour and who are discharged into the community or leave prior to receiving treatment. A history of suicide attempt is a robust predictor of repeat attempt and suicide death in Australia [3] and internationally [4, 5]. Risk of subsequent suicide death is highest within the first year of an index suicide attempt [6, 7] and up to six times higher among those who present to acute settings compared to the general population [8]. The heightened risk of suicide during the period immediately following discharge means that the acute setting is a critical point of intervention for those who are most at risk of subsequent suicide.

People who present to the acute settings for suicidal behaviour (including suicide ideation and suicide attempt) often have multiple psychological, social and interpersonal needs [9]. Apart from cognitive behavioural therapies, there is limited evidence for the effectiveness of psychosocial interventions on rates of suicidal behaviour following an episode of self-harm [10]. Although previous studies have shown people who receive some form of intervention, such as a psychosocial assessment [11–14], admission to a hospital bed [7, 15], or referral to outpatient follow-up care [6, 7] are less likely to engage in repeat suicidal behaviour or subsequently die by suicide, factors such as negative staff attitudes and stigma can impact patient engagement in follow-up services, as well as future help seeking behaviour [16–19]. Thus, even when patients receive assessment or referral to follow-up care, patients’ interactions and experience with healthcare staff can have a significant impact on their perception of care, and has the potential to mediate subsequent suicide risk.

Clinical Practice guidelines for the management of people who present to the acute settings for suicidal behaviour are available in Australia and New Zealand [20], Canada [21], the UK [22] and the US [23]. The purpose of these guidelines is to provide recommendations for the organisation of clinical services and management of medical and mental health. Nevertheless, these guidelines are subject to several limitations. For example, with the exception of the National Institute for Health and Care Excellence (NICE) in the UK [24, 25], few guidelines have been developed in consultation with consumers or people with lived experience of suicidal behaviour. The inclusion of consumers with lived experience is a quality indicator using the AGREE II (Appraisal of Guidelines Research and Evaluation II) instrument [26], is a key component of service planning, delivery and evaluation in the recent Fifth National Mental Health and Suicide Prevention Plan in Australia [27], and has been a policy directive in the National Health Services research and development process in the UK for the last two decades [28]. This approach, provides service users with lived experience the opportunity to identify existing gaps and identify services which may best meet their needs [28]. The inclusion of consumers with lived experience may be particularly relevant to the development of guidelines in the acute settings, given recent evidence that suggests those who have experienced a suicide attempt are often discharged from the acute settings without being offered the help they need [19].

In addition to the limited involvement of external stakeholders in the development of best practice recommendations for the management of suicidal behaviour in the acute settings, to date, existing guidelines have overwhelmingly focused on only a few aspects of the care continuum, from the point of presentation, triage, assessment, and discharge into the community. Consequently, current guidelines typically lack enough detail for implementation when faced with common organisational barriers in the acute settings such as high patient volumes, time pressures, availability of hospital beds, access to trained mental health consultants and presentation during afterhours [29–31]. For example, Clinical Practice Guidelines from both Australia and New Zealand [20] and the NICE guidelines in the UK [22] recommend that all people at risk of suicide who present to acute settings should receive a psychosocial assessment by a mental health consultant. But these guidelines do not describe what should be done in the absence of a mental health team and provide limited information on the content that should be included during the psychosocial assessment. It is precisely in these contexts, for example when there is limited access to trained mental health consultants, in which guidelines for the management of suicide risk may be most beneficial [30].

In an effort to further inform best practice, we conducted a Delphi expert consensus study to develop guidelines for suicide-related crisis response and aftercare in acute settings. The study comprised two panels. The first panel consisted of consumers with lived experience of suicidal behaviour, and the second consisted of professionals from acute settings, crisis and aftercare, healthcare, and suicide prevention sector. The Delphi method recruits panels of experts who make private ratings on statements until group consensus is met [32, 33]. In its original form, the Delphi method has been used in healthcare research to reach consensus when evidence is lacking, or when there is conflicting information [32]. Whilst there is some evidence for effectiveness of interventions for suicidal patients [34, 35], the present study aims to develop guidelines that take into account multiple aspects of the care continuum (i.e. from the point of presentation, triage, psychosocial assessment, discharge planning, and referral into the community), which as noted previously, are not routinely included in existing guidelines. For this reason, we chose the Delphi method in order to develop a comprehensive set of guidelines that take into account recommendations from the existing guideline literature and academic literature, while also assigning equal weight to the expert opinions of consumers and professionals. This method has been used previously to identify which approaches may be most beneficial, in the absence of empirical or conflicting evidence [33]; and has been broadly applied in the development of best practice guidelines [36–39].

## Methods

### The Delphi expert consensus method

Statements involving crisis response for people presenting to acute settings for suicidal behaviour were extracted from a search of scientific and grey literature, as well as interviews with key informants. The current study involved the following four phases: (i) a systematic search of peer-reviewed and grey literature; (ii) interviews with key informants; (iii) development of a questionnaire containing strategies for care of people who present for suicidal behaviour in acute settings, and; (iv) ratings by expert panel members of the strategies considered essential, or very important, to be included in the guidelines. The focus was on the organisation of services and the sequence of care rather than diagnostic decision-making. Where possible the guidelines have been reported in accordance with the quality standards outlined by the AGREE II [26] reporting checklist for clinical practice guidelines.

### Ethics, consent and permissions

All participants provided informed consent. Approval from the University of New South Wales Human Research Ethics Committee was obtained for the Delphi study (HC16632) and key informant interviews (HC16627).

### The systematic literature search

A systematic search was undertaken in order to generate statements that described responses that could be taken in acute settings to a person who presents for suicidal behaviour. We searched Medline, Embase, PsychINFO, Cochrane Reviews, and Web of Science from January 1, 2000 to October 21, 2016. Publications specific to guidelines, recommendation, policies or procedures for responding to self-harm and suicide in the emergency department or acute settings were identified (see Additional file 1 for the full search strategy). A grey literature search was also conducted in Google search engines to identify grey literature from websites, reports and online brochures. The first 50 sites for each Google search engine were examined for statements that referred to suicide response in the acute settings, using the search terms described above. Two reviewers (NTMH and LH) conducted initial eligibility screening based on title and abstract, followed by assessment of full-text versions. Sources were eligible for inclusion in the review if they contained statements which met the following criteria:

- Referred to people who have made a suicide attempt or engaged in self-harm, or referred to people who have experienced thoughts of suicide.
- Referred to an action performed by a member of staff in an acute setting or referred to a process or sequence of care initiated in an acute setting;

Studies were excluded if the article was published prior to the year 2000. Articles in languages other than English were excluded due to limitations in translation capacity among study authors. Both Australian and international literature were eligible for inclusion in the review.

### Questionnaire development and key informant interviews

A total of 1179 statements were initially extracted from the literature and key informant interviews. The statements were then screened for their content by two authors (NTMH and NR). Items that contained duplicate information (repeat themes and content) were excluded from the questionnaire. When two or more statements included duplicate information, but the role or person responsible for performing the action was incongruent, the authors flagged the statement for consideration by professionals working in suicide prevention crisis and aftercare services.

Interviews were conducted with key informants from public and district health networks in Australia, as well as with community health services involved in suicide crisis response and aftercare. Key informants were recruited from the same key organisations involved in the professional panel selection outlined below. The purpose of these interviews was to explore current crisis response and aftercare practices in acute settings, particularly those considered to be ‘best practice’. In total, 15 interviews were conducted with people working in crisis and aftercare. Action statements from the interviews were analysed and included in the questionnaire by two researchers (NTMH and NR).

The final questionnaire content was reviewed for comprehensibility and applicability to varied Australian acute settings by a small number of professional informants. These included a liaison psychiatrist, an academic researcher in suicide prevention, a person who provides suicide prevention training to emergency department personnel, and the director of a suicide prevention telephone crisis support service. The round one questionnaire consisted of 525 items divided into 11 sections (Table 1).

**Table 1:** Questionnaire sections and number of items

Section Name	Number of questions	Number of items^a^
Team roles	5	19
Initial contact	9	37
Comprehensive Psychosocial Assessment	6	77
Discharge care plan	16	66
Discharge	11	45
Referral	7	20
Follow-up	18	75
Staffing	4	21
Linkage with community services and aftercare	10	65
Training	11	82
Evaluation	3	18
Total	100	525

^a^Includes all listed items

### Panel formation

Two panels, one of health professionals and one of consumers, were invited to participate in the study. All panel members were Australian and aged 18 years or older. The panels are described below.

#### Health professional panel

Health professional panel members were recruited through Australian mental health and suicide prevention organisations (e.g. Suicide Prevention Australia, Mates in Construction, Lifeline Australia) as well as public health networks (e.g. the Australian Institute for Suicide Research and Prevention, Australian Suicide Prevention Advisory Council, Primary Healthcare Networks). Recruitment was limited to Australian organisations due to differences with health systems in other countries. Participants from the health professional panel were eligible for inclusion if they had: (a) clinical, research, or service provision experience with people experiencing suicide-related behaviour, and; (b) knowledge or contact with acute settings through service provision, employment, or client contact.

#### Consumer lived experience panel

The consumer ‘lived experience’ panel consisted of people with experience of suicidal ideation or behaviour, or who had been bereaved by suicide. Participants in the consumer panel were recruited from depression and mental health advocacy organisations, including ‘Beyondblue’s BlueVoices’, ‘Suicide Prevention Australia’s Lived Experience Network’, and ‘Being: Mental Health & Wellbeing Consumer Advisory Group’. Participants from the consumer panel were eligible for inclusion if they had: (a) experienced a suicide attempt or thoughts of suicide, or (b) had cared for a person who has made a suicide attempt or experienced thoughts of suicide, or (c) been bereaved by suicide. Participants from the lived experience panel were paid a small stipend for completion of each questionnaire.

### Delphi process

Panel members completed the questionnaires online using a web-based survey portal [40]. Panel members were asked to rate the importance of each item for inclusion in the guidelines using a five-point Likert scale, where items were rated as ‘1- essential’, ‘2 - important’, ‘3 - depends’, ‘4 - unimportant’, ‘5 - should not be included’. Panel members were also given the opportunity to provide feedback and suggest additional items at the end of each list and at the end of each section during the round one of the questionnaire.

After each rating round, panel members were provided with a summary of group ratings for consideration in the next rating round [33]. Items were re-rated in the next round if 80% or more of one panel endorsed an item as essential or important, or items were rated as either essential or important by 70–79% by both panel groups. Any items that did not meet the above conditions were excluded from the guidelines and subsequent rounds.

Responses from the first round were reviewed by two authors (NTMH and NR) who determined whether suggested items were new ideas not otherwise covered in the first questionnaire. Any item that was determined to be an original idea was included as a new item in the second round questionnaire. The second and third round questionnaires were administered four weeks apart. Participants were provided access to each questionnaire for three weeks during which they could edit their responses at any time. A copy of the round 1 Delphi questionnaire is shown the Additional file 2.

### Statistical analysis

On completion of each round, questionnaire responses were analysed for each item by obtaining the proportion of items rated as essential and important by both the professional panel and consumer panel, using the criteria noted above. Concordance rates between professional and consumer rated items were evaluated using Pearson’s correlation coefficient. Correlation coefficients of < 0.30, 0.30–50, and > 0.50 were considered small, medium and large, respectively. Post hoc analyses of attrition rates by employment were conducted in order to assess whether the diversity of panel members was maintained in each questionnaire round.

## Results

### Characteristics of participants

A total of 89 panel members participated in the study, including 50 consumers with lived experience, and 39 professionals (Table 2). The majority of participants were female (72 and 74% for the consumer and professional panel, respectively). The median age was 46 years (range 19–71) for the consumer panel, and 49 years (range 29–68) for the professional panel. The majority of participants in the professional panel were crisis and aftercare employees (including telephone counselling and crisis services and community and in home service workers, 24.4%), followed by mental health providers (20.5%), and acute settings employees (17.9%, Table 2).

**Table 2:** Number of participants in each questionnaire round

	Round 1	Round 2	Round 3
	N (%)	N (%)	N (%)
Consumer panel			
Personal experience of suicidal behaviour	29 (58.0)	25 (62.5)	24 (61.5)
Carer of someone with suicidal behaviour	11 (22.0)	8 (20.0	8 (20.5)
Both personal experience and carer	10 (20)	7 (17.5)	7 (17.9)
Professional panel			
Mental health provider^a^	8 (20.5)	7 (23.3)	6 (28.6)
Crisis and Aftercare service	10 (25.6)	8 (26.7)	3 (14.3)
Mental health nurse	5 (12.8)	3 (10.0)	1 (4.8)
Acute settings staff	7 (17.9)	5 (16.7)	5 (23.8)
Academic	3 (7.7)	3 (10.0)	3 (14.3)
Social worker	2 (5.1)	1 (3.3)	–
Primary care	1 (2.6)	1 (3.3)	1 (4.8)
Non-profit community suicide prevention	3 (7.7)	2 (6.7)	2 (9.5)
Sub Total	89 (100)	70 (76.9)^b^	60 (65.9)^b^

^a^Includes psychiatrist and psychologists

^b^Percent of round one participants

**Fig. 1:**
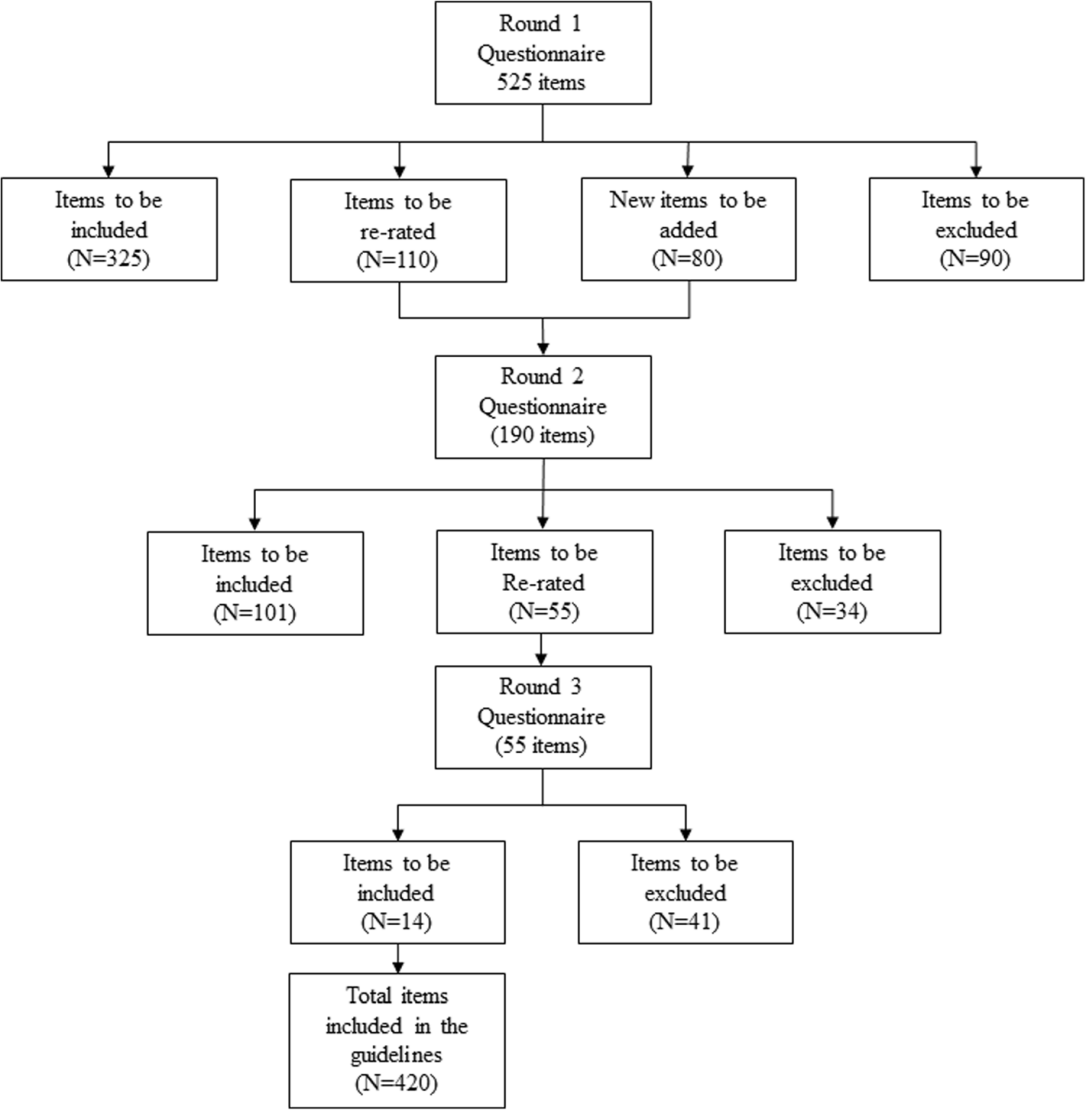
Flow diagram of item inclusion in each questionnaire round

The overall participation rate (those who participated in all three rounds) was 67.4% (78% consumers, 53.8% professionals, Table 2).The total number of items included, excluded, and re-rated in each round is shown in Fig. 1. Across the three rounds 420 items were rated ‘essential’ or ‘important’ for inclusion in the guidelines. Of those rated essential or important, four items were excluded following round one, as it was determined that items relating to special populations were outside the scope of the present study. Participants provided 763 feedback comments throughout the round one questionnaire. A total of 80 suggestions were incorporated into the second questionnaire as new items. The remaining suggestions consisted of justifications of participant’s selection of particular items, and were therefore excluded.

### Results of statements

There was a strong, positive correlation between ratings from the consumer and professional panels (*r* = 0.84, *p* < 0.01 and *r* = 0.69, p < 0.01, for the first and second questionnaires, respectively). The correlation between consumer and professional ratings was small but significant in the final questionnaire (*r* = 0.33, p < 0.01). Items that received notably higher endorsement by consumers included the provision of peer support, direct referral, communication between acute settings staff and services involved with the person’s ongoing care, assisting the person with finding support services that address lifestyle skills and domestic needs, and the availability of mental health staff during afterhours. Items that received higher endorsement by the professional panel included using video or teleconferencing to fill gaps in service provision; contact with the person’s next-of-kin, family, or professionals currently involved in the person’s treatment or care to identify additional information relevant to the comprehensive psychosocial assessment; and the assessment of risk factors such as the lethality of the suicide attempt.

Two of the authors (NTMH and NR) prepared the guidelines by grouping items by similar content under key headings. Items were edited into prose in order to provide the target audience with guidelines that retained the original wording, but were coherent and easy to read. The guidelines were provided to panel members for feedback. No changes to the guidelines were made. The final guidelines provide recommendations for integrated suicide-related crisis and follow-up care in emergency departments and other acute settings and are available in Additional file 3.

## Discussion

This study aimed to identify best practice strategies for crisis response and follow-up care in the acute settings for people who present for suicidal behaviour. The final guidelines consist of 420 items that outline the team structure; the provision of peer support; procedures and actions staff can take from initial contact, assessment, referral, discharge and follow-up; staff training; and linkage with community aftercare services. These items have been written into a set of guidelines that provide guidance to acute settings with respect to policy and practice [41]. In these guidelines, items were broadly grouped into areas of responsibility to ensure that the guidelines were targeted to the needs of different audiences. When responses between panels were compared, a higher proportion of consumers were more likely to endorse statements as essential or important when they involved non-clinical factors such as positive lifestyle skills, parenting skills, domestic support, harm minimisation plans, and the inclusion of peer support. In contrast, professionals were more likely to endorse items specific to assessing the lethality of the suicide attempt; the person’s involvement with the criminal or youth justice system; and items which included contacting the person’s carer, family, or friends as part of the psychosocial assessment process. This focus is more aligned with emergency medicine models that favour the identification and mitigation of short-term morbidity and mortality. Lower endorsement rates for some strategies, such as direct referral to aftercare services and the assessment of non-clinical factors may also reflect the difficulties health professionals experience in identifying appropriate aftercare services that meet the diverse needs of people who present to the acute settings for suicidal behaviour [9]. Nevertheless, inclusion of assessment of broader psychosocial factors in the current guidelines represents an important step in recognising and responding to the sometimes complex needs of individuals at risk of suicide who present to acute settings.

Participants’ feedback provided important insights into the rationale behind both consumers’ and professionals’ choices. When consumer feedback was incorporated into the second questionnaire, both panels were equally likely to endorse items as important or essential if they included the person’s consent. Similarly, the provision of brief discharge planning was included in the guidelines only when feedback involving contingent arrangements for more extensive aftercare planning in the community was incorporated into the second questionnaire. Feedback from the professionals showed that items that were judged as requiring significant allocation of financial resources were endorsed at a much lower rate, even if they were considered best practice. This may account for lower endorsement rates among the professional panel for statements involving the allocation of staff to accommodate suicide-related presentations during after-hours as well as higher endorsement rates among professionals for items that included the use of teleconferencing to fill provider gaps.

In contrast, feedback from the consumer panel emphasised the importance of empathy and compassion as well as concerns about stigma. The therapeutic value of empathy is a key theme in several qualitative studies [16, 17, 19, 42, 43] systematic reviews [18, 44] and clinical practice guidelines [20, 22]. For example, Taylor et al. [18] conducted a systematic review of qualitative studies investigating the healthcare experiences of people who have made a suicide attempt. In this review, the majority of studies that provided recommendations on service improvement identified a need for improved staff knowledge towards people who have self-harmed, as well as increased sympathy characterised by listening to patients and responding to them in a non-judgmental way. In a recent study of Australians who had experienced a suicide attempt, McKay et al. [42] found empathy was central to patients’ sense of support and care even among those who experienced ongoing suicidal ideation and vulnerability. In this study, empathy was characterised by the authors as ‘small kindnesses’ such as providing patients with privacy and a quiet place to rest. Whilst it is difficult to mandate empathy through clinical practice guidelines, results from the current Delphi study, and previous studies combined, suggest the attitudes of staff ought to be a priority for the response to suicidal behaviour in acute settings.

To the authors’ best knowledge, this is the first study that has used the Delphi method to develop best practice guidelines for the management of suicidal behaviour in acute settings. Particular strengths of the approach outlined here include the equal weight given to the views of people with lived experience. This is particularly relevant given consumer opinion is considered a key priority in healthcare policy directives in Australia and internationally, but is often overlooked in the delivery of health care responses. Further, the current guidelines address important gaps in the current guideline literature. For example they provide information on how to respond to people who present for suicidal behaviour in the absence of trained mental health staff and provide specific details on what constitutes a safe environment, as well as important considerations during assessment, referral and discharge.

### Limitations

Like all studies, the current study was subject to limitations. For example, although the study maintained the recommended minimum of 20 panel participants, a larger rate of attrition was observed in the professional panel. It is noteworthy that the final questionnaire consisted of seven out of eight professions included in the initial questionnaire, with the exception of participants from a social work background. As 87.5% of panel diversity was maintained, attrition was unlikely to bias optimum decision making processes essential to the Delphi consensus method [33].

Items included in the current guidelines were based on consensus between consumers with lived experience of suicidal behaviour, and a panel of professionals who have contact with people who have experienced suicidal behaviour. Given the focus on areas for which trial evidence is largely unavailable, Delphi study participant’s ratings are influenced by workplace culture and personal preferences. This is both a strength and a weakness of the methodology. Panel member ratings can be informed by their experience of what works in practice as well as by research evidence, although they may also be influenced by what is popular. In the present study, less than half of the professional panel comprised participants who have clinical experience working in the emergency department or other acute settings and it is possible that some panel members endorsed statements that were outside their expertise. Consequently, some items which were rated by participants as essential or important may not necessarily reflect the most up-to-date evidence. One such example is the inclusion of case management recommendations in the present guidelines. While there is evidence from some non-randomised trials that case-management, in addition to safety planning, may reduce repeat suicidal behaviour [45, 46], a recent Cochrane review of randomised controlled trials investigating the efficacy of psychosocial interventions following self-harm reported non-significant reductions in risk of repetition of self-harm and suicide death following case management [10, 33].

Lastly, a number of factors may account for the absence of items in the current guidelines. Included items reflect actions that both panels consider appropriate to be undertaken in most circumstances. Items that did not reach the threshold for consensus include those that are more likely to be rated ‘It depends’ which means that they may still be important or essential in a narrower range of circumstances. One such example is the exclusion of forensic history during the psychosocial assessment, which was included in the initial Round 1 survey (see additional file 2), but did not reach consensus between the two panels for inclusion in the guidelines. Thus, the absence of recommendations in the guidelines should not preclude acute settings providers from engaging in clinical decision-making that is not explicitly stated. This is particularly true in the management of complex mental health and medical presentations such as psychosis or patients affected by substances, which may require additional treatment in addition to the management of suicidal behaviour.

Although the current guidelines make important, practical, advances in comparison to previous guidelines, the effectiveness of the current guidelines in improving patient care for people who present for suicidal behaviour and reducing risk of re-attempt remains untested. There is a need for clear implementation strategies to ensure uptake and adherence to guidelines in acute settings. For example, these guidelines have been developed as part of the LifeSpan systems approach to suicide prevention (ACTRN12617000457347) and are a core component of the strategy aimed at Improving Emergency and Follow-Up Care for Suicidal Crisis. These guidelines are currently being implemented through a series of training webinars as part of the LifeSpan stepped-wedge randomized trial and are currently being used to audit local hospitals across four trial sites in NSW, Australia. These data will be used to evaluate the impact of these guidelines in order to determine their effectiveness as a stand-alone resource for mitigating suicide risk and re-attempt among high-risk individuals and will be reported elsewhere.

## Conclusion

These guidelines provide recommendations and strategies for responding to people who present for suicidal behaviour in acute settings based on the consensus of two expert panels comprising professional involved in the care of people who have experienced suicidal risk and people with lived experience. They provide recommendations that address some of the organisational barriers encountered by staff when working with suicidal persons, and give equal weight to the perspective of end users, whose experience with suicidal behaviour is a valuable source of expertise for informing evidence based practice. As with any guidelines, implementation will vary according to context and it is our hope that this research will be useful to the scientific and clinical community outside Australia. Future research should investigate ways to optimise implementation of these guidelines in order to improve equal access to quality care for people at risk of suicide who present to the acute settings.

## Additional files

Additional file 1: Full Search Strategy. (PDF 26 kb)

Additional file 2: Round 1. Delphi questionnaire. (PDF 177 kb)

Additional file 3: Guidelines for integrated suicide-related crisis and follow-up care in Emergency Departments and other acute settings. (PDF 2950 kb)
