# Supplementary material for: Development of best practice guidelines for suicide-related crisis response and aftercare in the emergency department or other acute settings: a Delphi expert consensus study
Source: BMC Psychiatry. 2019 Jan 7;19:6. doi: 10.1186/s12888-018-1995-1 (PMC6323675; doi:10.1186/s12888-018-1995-1)
Supplement: Supplementary file 1 — Full Search Strategy. (PDF 26 kb) [file 12888_2018_1995_MOESM1_ESM.pdf]

MEDLINE search strategy

1. (suicid* or self-harm or self-injur* or self-mutilation or parasuicide).ab,kw,ti.
2. (emergency center or emergency centre or emergency admission or emergency presentation or emergency medicine or emergency department or emergency unit or emergency room or emergency response or emergency physician).ab,kw,ti.
3. (trauma centre or trauma center).ab,kw,ti.
4. (hospital or triage or discharge or liason or acute).ab,kw,ti.
5. (accident and emergency).ab,kw,ti
6. (guide* or policy or recommend* or coodinat* or integrat* or collaborat* or protocol).ab,kw,ti.
7. 2 or 3 or 4 or 5
8. 1 and 7 and 6
