# Supplementary material for: Development of best practice guidelines for suicide-related crisis response and aftercare in the emergency department or other acute settings: a Delphi expert consensus study
Source: BMC Psychiatry. 2019 Jan 7;19:6. doi: 10.1186/s12888-018-1995-1 (PMC6323675; doi:10.1186/s12888-018-1995-1)
Supplement: Supplementary file 2 — Round 1. Delphi questionnaire. (PDF 177 kb) [file 12888_2018_1995_MOESM2_ESM.pdf]

Additional File 2

Round 1. Delphi questionnaire: Development of best practice guidelines in crisis and suicide prevention

Definition of terms used in this survey

Unless otherwise specified, **'the person'** OR '**the person at risk of suicide'**refers to a person who has made a suicide attempt or is experiencing thoughts of suicide.

Acute setting is the term used to refer to settings in which people at risk of suicide receive active but short-term treatment. In this context they include the Emergency Department and Psychiatric Emergency Care Centres.

The Suicide Response Team (SRT) refers to members of the hospital Mental Health Team responsible for the coordinated response to suicide presentations. The team may comprise multiple members with designated roles, or a single clinician, such as a mental health nurse.

Comprehensive Psychosocial Assessment refers to the evaluation of a person's mental, physical and emotional health, as well as their ability to function in the community. This assessment plays a central role in helping to improve the aspects of the person's life that have contributed to their risk of suicide.

Section 1: Team roles

The Suicide Response Team (SRT)

1. The member of staff who is responsible for overseeing the operations of the mental health team should ensure all SRT members receive appropriate training in the care of a person at risk of suicide.
2. The SRT should have members co-located in the department where people at risk of suicide receive acute care.
3. A person from the SRT should act as a single point of contact for referrals from the acute setting.
4. The SRT should include the following staff roles:
    - A liaison psychiatrist
    - A member of staff trained in delivery of psychosocial assessments
    - A member of staff in crisis intervention
    - A member of staff in internal and external referrals
    - A member of staff in follow-up care
    - A member of staff trained in the formulation of a discharge care plan
    - A person responsible for discharge and transfer of care
    - A person that provides follow-up

Acute settings without a staffed SRT

5. The member of staff who is responsible for coordinating the mental health team should arrange the following services when SRT staff are not available:
    - Provision of a 24-hour area mental health service telephone triage that offers instructions and advice to before people presenting to the acute setting.
    - Partnerships with allied health or ambulatory care services.
    - Partnerships with community-based services (e.g., local NGO's, outpatient services, and social services).
    - Liaison with psychiatrists via videoconference for assessments and consultation.
    - Co-location of staff from partner agencies in the acute setting to assist with care coordination.
    - Contracting staff with the relevant skills and experience to provide coordination in the acute setting (e.g., Sub-contracting from community agencies).
    - Links to a community-based suicide prevention team to assist with aftercare and follow-up services.

Section 2: Presenting to the acute setting

Initial contact

Initial contact is the first point of contact with medical services made by a person at risk of suicide, a person's carer, or clinical practitioner.

6. The person presenting for a suicide attempt or thoughts of suicide should be triaged directly to the SRT.

Provision of peer support

7. During initial presentation staff from the acute setting staff or SRT should arrange a dedicated peer support worker to support the person at risk of suicide.
8. The trained peer support worker should be:
    - A volunteer
    - A paid hospital employee
    - A person from a partnering community agency
9. The peer support worker should:
    - Assist the person presenting for suicidality in the acute setting
    - Ensure the person is treated with dignity and respect
    - Assist the person to understand hospital policies and procedures

- Share their own stories of recovery as a means of problem solving and inspiring hope
- Accompany the person while they wait for consultation with the SRT
- Provide basic physical comforts such as blankets, towels, meal trays, reading materials, and water

10. Upon referral the SRT should:
    - Notify key hospital workers or case-managers previously involved with the person's care.
    - Link the person to hospital services that address comorbidities (e.g., Drug and alcohol services).
    - Refer the person to inpatient care when necessary (e.g., in cases of people with command hallucinations or ready access to lethal means).
    - Follow-up people transferred to different hospital wards (e.g., Following surgical intervention) to ensure they receive a comprehensive psychosocial assessment.
    - Provide emotional support and help to carers accompanying the person and who are experiencing high levels of stress and anxiety.
    - Provide carers with information about caring for someone with suicidality
    - Provide referrals for carers to local services and support groups.
    - Identify people who frequently attend the acute setting
    - Arrange the comprehensive psychosocial assessment
    - Complete discharge care planning
    - Refer the person to aftercare services

11. The SRT should request consent for the following procedures:
    - Follow-up and aftercare contact from a designated person from the SRT or relevant community service.
    - Leaving a phone message.
    - Sharing information from the Comprehensive Psychosocial Assessment to coordinate the person's aftercare.
    - Sharing the person's information with aftercare services involved with their care.
    - Contacting other people who may be involved in the person's care (friends, family, current physicians) for corroborative information during the comprehensive psychosocial assessment.

Written information provided in the acute setting

12. The SRT should ensure that written information on the following topics is available waiting for consultation with the SRT:
    - Referral and aftercare services available at the hospital and in the community.

- Alternatives to inpatient admission (e.g., community crisis houses, day hospitals, home treatment, respite and other relevant local community programs).
- A person's rights in healthcare
- Skills and education-based workshops
- Community support groups
- Available mental health crisis services (e.g., mental health drop in clinics).
- Any non-clinical services that may alleviate isolation and promote hope

Providing a safe environment

13. Staff in acute settings should ensure that the environment is safe by providing:
    - A private and comfortable place for person to sit and wait for consultation
    - A private and comfortable place to complete the Comprehensive Psychosocial Assessment
    - CCTV monitoring
    - A signed and dated environmental safety audit kept on record in the acute settings.
14. During initial contact staff should offer nicotine replacement options to people at risk of suicide who are nicotine dependent to help alleviate additional stress

Section 3: Comprehensive Psychosocial Assessment

15. The SRT should administer a comprehensive psychosocial assessment to all people who are referred to the SRT
16. The SRT will assess the needs of carers accompanying the person
17. If the SRT cannot complete a comprehensive psychosocial assessment due to staff unavailability, or refusal by the person, the following should occur:
    - Acute setting staff should permit and encourage admission for a 24 to 48-hour period of hospitalization during which time the assessment can be carried out.
        - The SRT liaison psychiatrist should assess the person's mental capacity.
        - The SRT should arrange the comprehensive psychosocial assessment to be performed via remote video or teleconferencing.
        - The SRT should refer a person to an appropriate community-based service for the comprehensive psychosocial assessment.
        - The SRT will ask treating health professionals and people in the person's social network to provide information on the following:
        - Their beliefs about the current presentation
        - Behavioural changes in the person
        - Access to means of suicide such as firearms or stockpiles of medication

- The SRT will use the results from the comprehensive psychosocial assessment to complete the following:
- Provide a diagnosis
- Develop a treatment care plan
- Provide education about the person's condition and treatment options
- Provide a referral for psychiatric consultation, if applicable.
- Provide referrals to other hospital or outpatient services such as mental health treatment, substance abuse treatment, family counselling and other social services etc.
- Educate family members or carers about the person's condition and treatment options

18. The comprehensive psychosocial assessment should cover:
    - The physical injury and its severity (e.g., Self-poisoning, laceration)
    - The motives underlying the suicide attempt
    - The circumstances that led to the suicide attempt including suicidal ideation and persistence of ideation
    - Medication and substance use history
    - Perceived burden on others
    - Immediate mental health needs
    - Medium term mental health needs
    - Long term mental health needs
    - Immediate social needs
    - Medium term social needs
    - Long term social needs
    - Whether the person is accompanied or unaccompanied
    - A review of mental health symptoms
    - Stressors that the person is subject to
    - The ability of the person to enter into a therapeutic alliance/partnership
    - Problem-solving strategies the person is open to
    - The person's acknowledgement of self-destructive behaviours
    - The person's ability to seek and access help and identify any barriers to accessing services
    - Trauma history and treatment needs
    - Underlying psychiatric or medical conditions and medical history
    - Suicide attempt and self-harm history
    - Exposure to someone else's suicidal behaviour or suicide death
    - The potential lethality of the chosen method
    - Evidence of preparatory behaviours and planned precautions to prevent discovery or interference of the person's suicide attempt
    - Ambivalence about living or dying
    - Evidence of covert suicide ideation (e.g., Making a will, paying debts, hinting – "you will not have to worry about me any more")
    - Whether the person has dependents
    - An assessment of negative feelings including depression, hopelessness,

helplessness, loneliness, feeling trapped, and continuing suicidal intent
- Behavioural observations including whether the person is quiet, withdrawn or difficult to engage
- Whether the person is unable/unwilling to communicate (not due to language barrier)
- An assessment of impulsiveness and risk taking behaviour
- Personality style
- Exposure to domestic violence, neglect or abuse
- A comprehensive mental state examination
- The assessment of basic cognitive testing to account for the possibility of cognitive impairment as either the result of the suicide attempt or an underlying condition
- Core values/beliefs, goals and strengths
- The person's positive coping and problem solving skills
- The person's support resources
- The person’s concerns about stigma
- Financial barriers
- Education needs (for those in education)
- Life skill needs
- Parenting skills (for those with children)
- Immediate supports
- Engagement with help
- Religion considerations
- Dietary considerations
- Veteran status and war trauma
- Involvement with the criminal or youth justice systems
- An assessment of family and social connectedness
- Involvement with the criminal or youth justice systems
- History of problem gambling
- Ability to maintain hygiene and bodily functions
- Ability to fulfill family and occupational responsibilities
- Ability to maintain sufficient hydration and nutrition,
- Ability to interact with others.
- Assessment of protective factors, e.g., Family support, coping skills
- Psychological first aid, including problem solving counseling

19. The SRT should follow the same principles as for the assessment of older adults, but also include the following:
    - Assess for cognitive impairment.
    - Assess meaningful activities that the person engages in (e.g., religious practices, integration in social networks and clubs, having a hobby).
    - Assess possible sensory impairment and communication problems.
    - Assess functional status, e.g., activities of daily living
20. Where a clinical decision results in the person going home, the SRT should organise a follow-up assessment through specialised aged psychiatry services or care coordination programs.

Section 4: The discharge care plan

The main aim of the Discharge Care Plan (DCP) is to ensure a safe and successful transition for the person from the acute setting to the community. It provides a means of synthesising assessment information and agreed strategies and is particularly important for people with multiple and complex needs. It specifies the steps that must be taken and assists the person to come to a decision that is appropriate for their needs, wishes, values and circumstances.

21. The DCP should be based on:
    - Underlying factors that impact the severity of symptoms at different points in time and during crisis.
    - Culturally appropriate assessments and referrals.
22. The person's capacity for decision-making will determine their level of involvement with treatment planning.
23. The SRT should provide the DCP to the following people:
    - Everyone who presents to the acute setting and is at risk of suicide.
    - A person who frequently attends the acute setting and is at risk of suicide.
    - A person who has established contact or treatment with community-based services (eg. Community mental health case management).
    - A person with an outpatient appointment (e.g., A person on the waitlist for a psychotherapy appointment).
    - A person at risk of re-presenting to the acute setting.

Development of the DCP

24. The SRT works with the person to develop the DCP.
25. The DCP should contain strategies that are accessible, available and valued by the person, their family and/or carer.
26. The DCP includes:
    - Developmentally appropriate language and information.
    - Mental health symptoms.
    - Instructions for the person's medication including frequency, dosage and side effects
    - A schedule of appointments for follow-up and aftercare
    - Contact details for all referred aftercare services (eg. psychosocial, psychological and medical, and community service based)
    - 24/7 mental health contact details (including phone support services such as Lifeline)
    - Contact details for crisis assistance
    - Contact details for community mental health services
    - Arrangements to overcome barriers to accessing aftercare services
    - Follow-up procedures following non-compliance or failure to attend aftercare appointments.

- Recommendations and actions that address needs identified in the comprehensive psychosocial assessment.
- Specific steps to seek help and support if symptoms re-occur or worsen or the situation deteriorates following discharge.
- Education about warning signs of possible relapse and what to do
- Details of the person's nominated support network and related contact details
- Recommendations to engage with the persons support network
- Recommendations to reduce social isolation
- Contingency arrangements for contacting specialists.
- A Harm minimization plan for alcohol and drug use.
- Education on positive lifestyle skills, including, maintenance of positive affect, and healthy sleep.
- The persons current motivations for change
- Strategies to mitigate intolerable distress, pain, coping, and suicidal thoughts at home.
- Housing support recommendations and action needed to secure accommodation (if applicable).
- Domestic support recommendations and arrangements
- Vocational support recommendations and arrangements
- Employment support recommendations and arrangements
- Education support recommendations and arrangements
- Training support recommendations and arrangements
- Disability support recommendations and arrangements
- Income support recommendations and arrangements
- Cultural and faith recommendations and arrangements
- Therapeutic leisure recommendations and arrangements
- Recommendations that promote independence
- Education support recommendations and arrangements
- Life skills support recommendations and arrangements
- Parenting skills recommendations and arrangements
- Positive risk-taking approaches

Provision of the DCP to the person

27. The SRT should provide a comprehensive DCP to the person before they are discharged or transferred from the acute setting.
28. The SRT should provide a brief DCP to the person if:
    - Arrangements have been made for the community mental health team to complete care planning with the person during a follow-up appointment.

- Arrangements have been made for the SRT to contact the person and develop the care plan in the community during a scheduled appointment.

29. The SRT should make arrangements to ensure all people who receive a brief DCP are provided a more extensive DCP as follows:
    - Within 24 hours of discharge
    - Within 7 days of discharge.
30. The SRT will engage in more intensive discharge planning for people with multiple needs identified in the psychosocial assessment.
31. The SRT will determine whether a community treatment order will be sought for non-adherence to the DCP.
32. People who frequently attend the acute setting will be encouraged to develop a crisis management plan (Documents that set out a person's preferences for future medical treatment which gives the person the opportunity to work with health professionals to decide, when they are well, on the type of treatment they would like to receive in a time of crisis).
33. The SRT will include elements in the DCP contrary to the person's preferences if:
    - The person lacks decision-making capacity around those elements
    - The elements represent the best practically available management option
    - The elements are the least restrictive of the person's freedom
34. The SRT should:
    - Provide the person with DCP in written format
    - Provide the DCP verbally to the person
    - Arrange for the DCP to be signed off by the person
    - Provide the DCP in other delivery format options determined by the person (email, mail).
35. The SRT will provide a written copy of the DCP to the following people:
    - The person
    - Carer/guardian/next-of-kin
    - The person's GP within 24 hours of the discharge
    - Agencies/services referred to provide aftercare to the person
36. The SRT will only provide aftercare services with information from DCP that is relevant to the service.

Section 5: Discharge

37. The SRT should carry out the following activities during discharge:
    - Notifies current community-based services utilised by the person
    - Arrange any outpatient services that the person currently receives to be suspended while the person receives inpatient or sub-acute treatment.
    - Assists in finding a substitute setting for the person including inpatient, sub-acute or private hospital bed, respite care or special accommodation

- Assists with admission or transfer of the person to an alternate setting, if applicable.
- Notifies the person's GP
- Arranges support for dependents and carers
- Exchanges information between service providers (with the person’s consent)
- Arranges reviews and reassessments.
- Provides handover to medical, nursing and allied health practitioners in the relevant inpatient or sub-acute areas on transfer of the person.
- Provides all acute setting notes and a copy of investigation results to the corresponding facility for all people being transferred to acute or inpatient mental health care.

38. For a person eligible for discharge, the SRT ensures that:
    - The person has received the DCP
    - The contact details of the person, their GP, as well as those supporting them in the community, are updated in the hospitals electronic records The person has received medication and a prescription supply, if applicable
    - The person has received information stating the benefits of follow up and treatment adherence
    - The person has received information such as crisis cards, business cards, and brochures from community services and partners.
    - The person has adequate arrangements to be transported to a safe location at discharge.
    - Phone hand-over to the relevant agency (during regular business hours) has been performed.
39. If possible, the SRT ensures communication with services involved in the person’s aftercare is made before the person is discharged.
40. The SRT ensures the person's carer /guardian/next of kin is provided with discharge information.
41. The SRT ensures the person's carer /guardian/next of kin is provided with discharge information **with the persons consent.**
42. The person arranging discharge will document whether the person has given consent for their discharge information to be shared with the person’s carer or support person.
43. Discharge information provided to the persons carer or support person includes the following:
    - Safety measure advice including means restriction counselling (i.e. Securing medications)
    - Harm minimisation techniques
    - Healthy strategies to help with person cope with distress
    - Encouragement to support the person being discharged
    - Encouragement to accompany the person to aftercare appointments
    - Specific risks related to the person
    - Crisis and emergency contact numbers
    - Explicit contingency arrangements so the carer can contact specialist services if they need to.

- Advice about how to handle situations in which the person is unwell but avoiding or resisting help
- Maintaining appropriate supervision by knowing **where** the person is at all times
- Maintaining appropriate supervision by knowing **who** the person is with at all times.

44. The SRT provides a discharge summary that includes:
    - Recommendations to the person's GP if the person needs additional help.
    - The person's condition/diagnosis/crisis
    - Reason for attending the acute setting
    - Summary of the assessments
    - Summary of the DCP
    - Interventions arranged
    - Interventions undertaken by the SRT
45. The SRT provides the discharge summary within 24 hours of discharge.
46. The SRT provides the discharge summary to the following people:
    - The person
    - The person's GP
    - The person/service responsible for follow-up
    - All services involved with the person's aftercare (including follow-up and outpatient services)
47. The person can request the discharge summary is provided in a format best suited to them (e.g., Email)

Section 6: Referral

Referral describes the transmission of a person's personal or health information between one agency and another with a request for further assessment, care or treatment.

48. The SRT ensures that aftercare referrals are established as follows:
    - Within 7 days of discharge
    - No later than the first workday after discharge.
    - Within 24- 72 hours for **all** cases
49. The SRT will contact the relevant agency prior to making a referral to ensure the agency can accommodate the timely implementation of the referral request.
50. Referrals should be prioritised as follows:
    - Low – meaning hold over during peak demand' or respond within seven working days of obtaining consumer consent
    - Routine – meaning 'attend to in date order' and may include a person being placed on a waiting list or respond within seven working days of obtaining the persons consent

- Urgent – meaning referral 'cannot wait' and must occur:
    i. The next day
    ii. Within two working days of discharge.

51. The SRT is responsible for the following:
    - Confirming that the person's referral has been received by the relevant aftercare service
    - Documenting referral outcomes
    - Providing referrals for support services to the person's carer or next of kin
    - Postvention and bereavement aftercare for carers, family and friends of a person who died by suicide when entering the acute setting.
    - Arranging a follow-up referral to the person's GP
52. The SRT should arrange aftercare using the following referral methods:
    - e-Referral
    - A standard referral form that is accepted between all community aftercare services that have linkage agreements with the acute setting
    - A brokerage system where one health professional refers to an agency which then allocates a referral to another health professional.
    - A register system where lists of available health providers are offered to referring practitioners.
    - Direct referral where one health professional refers a consumer directly to another.
53. The SRT should have a single point of contact for community referrals to aftercare services.
54. An SRT staff member should provide referrals for carers to local services and support groups.

Section 7: Follow-up

Follow-up refers to a systematic process of monitoring and reviewing care plans, referrals and interventions provided through by the SRT.

55. The SRT should arrange follow-up services to **everyone** who presented to the acute setting having made a suicide attempt or experiencing thoughts of suicide.
56. The SRT should arrange follow-up services to people based on the needs identified by the comprehensive psychosocial assessment.
57. The staff member responsible for follow-up should have access to the patient administration systems that contain information about people requiring follow-up.
58. The SRT should actively case manage the person during follow-up.
59. The SRT should arrange for active case management of the person during follow-up.
60. Case management consists of the following:
    - Explaining the purpose of the follow-up contact.

- Explaining follow-up is time limited and is not designed to replace short-term treatment
- Explaining when the schedule of follow-up contact will end
- Determining whether treatment/case management has been sought, organised, and delivered.
- Expressing concern and support.
- Providing telephone reminders of appointments.
- Facilitate engagement with relevant services
- Including additional referrals to community supports, when applicable.
- Encouraging the people who have dropped-out to return to treatment.
- Encouragement to seek treatment
- Discussing the person's progress against their DCP
- Assessing whether the DCP has been useful
- Establishing a more suitable therapeutic plan in collaboration with the person and health services (if applicable).
- Mood check
- Discussing solutions to problems, if applicable
- Reviewing barriers to treatment and adherence and develop alternative strategies
- Discussing the management of work
- Discussing the management of stressors
- Assessing the success of the person's transition back into the community.
- Invite the person to stay in touch and call whenever they feel they are in crisis. Discussing future management planning
- Motivational counselling
- Liaising with all service providers to ensure referred aftercare has been received.

61. There should be a process of asking people with lived experience for feedback on their service experience.
62. The SRT should classify some people as requiring brief follow-up while others require assertive follow-up.
63. During brief follow-up the SRT should monitor the person's engagement with aftercare services by making direct contact with the following:
    - The person's treating GP
    - The persons carer
    - Next-of-kin
    - The person's family/relatives
    - Relevant outpatient providers in the community
64. People eligible for assertive follow-up include:

- People with **diagnosed** mental illness (i.e. Depression, schizophrenia, bipolar etc.)
- People with **suspected** mental illness (i.e. Depression, schizophrenia, bipolar etc.)
- People with a history of poor medication adherence
- People with a history of poor service/treatment adherence.

65. Assertive follow-up includes:
    - More frequent contact
    - Home-visits by a member of the SRT or community agency
    - Intensive case management
    - Aged-care assessment service
    - Out-reach support program
    - Home assessment rehabilitation team
    - Aged care team support services
66. The SRT should provide follow-up using the following methods:
    - A visit by a practitioner to the person's place of residence
    - Letters of support
    - Face-to-face
    - Telephone
    - Videoconference
    - Text messages used to schedule a date and time for upcoming and subsequent follow-up appointments
    - Email
    - 'Crisis cards' with emergency phone numbers and safety measures
    - Post cards with care messages
67. First follow-up contact should occur within the following timeframe:
    - Within 24 hours for people requiring assertive follow-up
    - Within 24 hours for all people
    - Within 72 hours for all people
68. The second follow-up appointment will occur at seven days, and then at monthly intervals
69. The SRT will complete a maximum of 3 contacts with the discharged person.
70. Frequency of follow-up should be revised with each visit.
71. The SRT should cease follow-up when:
    - Treatment/case management has been organised for people who received aftercare referrals from the acute setting
    - The person has received treatment from the referred service since discharge from the acute setting.
72. A reminder system should be in place for people discharged from the acute setting.
73. The SRT should arrange a home visit for people who miss follow-up appointments.

Section 8: Staffing

74. In order to understand when rates of presentation are highest in the community among people who have made a suicide attempt or are experiencing thoughts of suicide a designated member of staff in the acute setting should be responsible for collecting the following data:
    - Number of presentations
    - Time of presentation
    - Day of presentation
    - Time the person was seen by the SRT or other mental health staff where there is no SRT available
    - Number of people who did not wait for treatment
    - Number of psychiatric or acute beds
75. The manager responsible for staffing should accommodate for periods when known demand is high by increasing the number of staff scheduled.
76. The manager responsible for staffing should ensure the following:
    - The SRT will be staffed 24 hours, at all times.
    - The number of mental health clinicians co-located in the acute setting should be proportional to the overall number of yearly presentations by people who have made a suicide attempt or are experiencing thoughts of suicide.
    - A liaison psychiatrist should be available at all times in person
    - A liaison psychiatrist should be available at all times by teleconferencing
    - Caseworkers from the SRT should be available during after hours to respond to crises.
77. The member of staff responsible for staff scheduling (also known as the staff roster) should ensure the hospital without a SRT is staffed by doing the following:
    - Hiring additional providers who have the clinical skills and experience to work with people who are at risk of suicide.
    - During periods of high demand, allocating providers from other departments in the hospital or health care system with the skills and experience to assess and treat people by people who have made suicide attempts or are experiencing thoughts of suicide.
    - Using teleconferencing services for consultation with liaison psychiatry services at alternate hospitals
    - Contracting agencies in the community who can provide services such as monitoring, assessment, referrals and follow-up
    - Ensuring that a psychiatry register or psychiatry liaison is available on call, at all times
    - Ensuring that an after hours doctor is available to assess people at risk of suicide presenting to the acute setting outside regular hours

- Ensuring that an after hours mental health nurse is available to assess people at risk of suicide presenting to the acute setting outside regular hours.
- Ensuring that an after hours social worker is available to assess people at risk of suicide presenting to the acute setting outside regular hours.
- Ensuring that a person trained in comprehensive psychosocial assessment, supportive counselling and intervention is available 24/7.

Section 9: Linkage with community services and aftercare

Community mental health service components include urgent community-based assessment and short-term treatment interventions for people with mental illness in crisis, intensive long-term support for people with prolonged and severe mental illness and associated high-level disability, and non-urgent continuing care services for people with mental illness, and their families or carers in the community.

78. The SRT should arrange for inter-agency protocols that link the acute setting to community agencies, NGOs and Community services.
79. The interagency protocol should aim to facilitate the following:
    - An agreement to allow some people to receive priority treatment when referred from the acute setting based on clinical need.
    - Document the responsibilities of each aftercare service when the person has multiple aftercare needs
    - An agreement for accessing secondary consultation on request
    - Aftercare referral arrangements
    - An agreement between community agencies that people with certain comorbidities (e.g., alcohol misuse) will not be excluded from aftercare services.
    - Follow-up arrangements
    - Treatment arrangements
    - Assessment arrangements
    - Shared care arrangements
    - Information sharing arrangements
    - Access to clinical mediation and advocacy (e.g., social workers in aged care that can assist the individual with deciding treatment options).
    - Outreach arrangements for hospitals that lack the capacity to employ in-house services.
80. The SRT should facilitates collaboration and coordination across health and social care through the following mechanisms:
    - Development of a standardised form for communicating with partnering community services
    - The development of a formal agreement through a memorandum of understanding

- Incorporating healthcare information exchanges to exchange records and coordinate services with community partners
- Providing joint education opportunities such as coordination of interprofessional education programs
- Inviting different providers to present at formal professional development events
- Shared records
- Mentoring
- Use of telepsychiatry to close provider gaps if local resources are limited.
- Provide training in risk management
- Facilitate feedback and communication streams for service issues
- Allocation of time in the acute setting for designated mental health providers from other hospital departments to assess and treat a person presenting for suicide behaviour.
- Provide opportunities for joint clinical rounds between hospital services involved in the persons care
- Share information via common electronic medical records
- Providing efficient and standardised data collection across sectors
- The use of registries to support population based care
- The co-location of outpatient services in close proximity to the acute setting
- Provide routine screening for dual diagnosis across sectors
- Cross-sector rotations
- Provide adequate space and resources (rooms, computers) for co-located services.
- Acute setting staff will provide other hospital department services (e.g., social workers, sexual health, drug and alcohol) with a copy of the admission list to identify people who previously or frequently attend the service.
- The use of online models such as health information exchanges that help providers exchange records and coordinate services
- The facilitation of joint staffing meetings with inpatient units and acute psychiatric care departments.

81. The SRT should develop and maintain a resource manual of local outpatient mental health providers and community services to facilitate continuity of care.
82. The resource manual is used to coordinate the following:
    - Referral
    - DCP
    - Follow-up
    - Continuity of care
83. The resource manual includes the following:
    - A list of evidence-based services.

- A description of the service provided
- A description of eligibility criteria
- Relevant skills of the agency/service
- Specialisations of the service provided
- Contact information
- Referral information

84. The SRT will host a regular memorandum of understanding meeting or case review meeting to discuss the management of people who have attempted suicide.
85. The SRT should invite the following people to attend the memorandum of understanding meeting:
    - Key agency representatives involved with the treatment and aftercare
    - Ambulance staff
    - Police
    - Security
    - Hospital staff
    - Community mental health staff
    - Trained Peer support workers.
86. The meeting will include the following:
    - Clear terms of participation
    - Identification of repeat attenders
    - Identification of those at risk to loss of contact
    - Review of the discharged person's progress
    - Referrals
    - Follow-up
    - Transfer of care
    - Transport issues
    - Acute behaviour
    - Aftercare services
87. The SRT should assign staff to the local suicide prevention network collaboration meetings when one exists.

Section 10: Training

88. The following people should be made accountable to ensure all staff members who have contact with a person who has made a suicide attempt or has thoughts of suicide:
    - Nursing Directors
    - The hospital executive
    - Other
89. The manager responsible for training and education in the acute setting will assess the training needs of staff

90. The manager responsible for training and education will ensure that all training is in accordance with evidence-based practice.
91. The manager responsible for training and education will provide training to the following:
    - All staff in the acute setting who have contact with people who have made a suicide attempt or are experiencing thoughts of suicide.
    - Paramedics (including ambulance staff)
    - Social workers
    - Youth worker
    - Acute setting security
    - Other allied services that attend to person's in the acute setting
92. The training will cover:
    - Understanding the complex causes of suicidality and mental illness
    - The importance of empathy
    - The effects of suicidality and mental illness
    - The location of psychiatry services at all hours of operation
93. In acute settings without a psychiatric liaison team, the manager responsible for training and education will ensure that staff receives more comprehensive training in suicide and mental health.
94. The manager responsible for training and education in the acute setting will ensure that staff members working with people with suicidal behaviour have the opportunity for:
    - Supervision
    - Peer discussion and support
    - Avenues to discuss and understand their own reactions
    - Case review meetings and opportunity for supervision.
    - Access to appropriate clinical supervision, consultation or advice from a senior clinician at all times.
95. The SRT will demonstrate the following training core competencies:
    - Specific requirements of common law and legislation.
    - How to respond respectfully, in a non-stigmatising, non-discriminatory manner
    - Recognising differing presentations of possible suicidal behaviour in different age groups
    - Undertaking detailed evaluations of suicidal behaviour and ideation
    - Undertaking mental state assessments
    - Undertaking comprehensive psychosocial assessments
    - Undertaking culturally relevant and sensitive assessments
    - Basic understanding of medico-legal issues in the delivery of mental healthcare
    - Diagnostic formulation

- Generating and implementing management/care plans
- Assessing hostile or guarded people
- Knowledge of sequence of care upon presentation to the acute setting.
- Providing solution focused therapy training
- Using corroborative information to aid in diagnosis, assessment, management, and discharge planning by confirming the person's history from other sources such as the patient's medical file, carers, family, GP, Case Manager, Police, Ambulance, other clinicians or support service providers.
- A detailed understanding of all local, evidence based, resources relevant to the support of service users
- The skills to formally assess a person's decision-making capacity when they disagree with treatment recommendations or if they decide to leave the acute setting before completion of the assessment.
- Procedures to notify police of firearms/weapons present in the home, when applicable.
- Skills in person-centred engagement
- Building a therapeutic alliance
- Implementation of hospital policies, protocols and guidelines
- Reflective practice
- The impact of emotions and feelings on interactions with others
- Regulating emotions and feelings
- How to identify comorbidity and its impact (e.g., alcohol and drug disorders)
- Procedures for engaging partnering services who have shared governance of the person's care.
- Positive risk-taking strategies
- How to adequately inform people of their treatment options, particularly when they have reduced judgment
- Listening and talking to the patient, explaining actions, and providing reassurance.
- Engaging the person as partner in the design of their care
- Reducing the noise from the environment if possible
- Problem solving techniques
- Alcohol problems including brief interventions and detoxification
- Management of frequent attenders
- Managing acute behavioural disturbance
- Managing self-harm
- Managing treatment adherence
- Mental Health Triage
- The assessment of mental capacity and its application in the acute setting

- The impact of cultural differences on influencing symptomatology, perception of symptoms, help seeking behaviour, and clinical judgment
- Procedures for contacting child protection services
- Means restriction counselling which informs the person’s carer/next of kin/ guardian about the dangers of access to medication, and other lethal objects
- Stressors that lead people to suicide
- The impact of attitudes and judgments on help-seeking behaviour
- Ways to make people feel validated and listened to
- Behaviour that offers comfort, reassurance and hope
- How to train peer support workers to assist and advocate for people experiencing a suicidality in the acute setting

96. The SRT will ensure the following is documented:
    - The person's triage status
    - The Psychosocial assessment
    - Sources of corroborative history
    - DCP
    - Discharge letter
    - Referrals
    - Assessment of risk
    - Mental state
    - Follow-up
    - Actions and precautions taken as an outcome of those assessments
    - Consultation with supervisors
    - Consultation with a person's key carer network
    - Ongoing communication across the care system
    - Treatment
    - Reasons for refusal of treatment (if this has occurred).
97. All Correspondence with partnering agencies should occur via by secure fax or secure email.

Section 11: Evaluation

98. There should be member of staff in the acute setting responsible for implementing a mental health data collection and monitoring framework that assesses service activity and quality.
99. The SRT will keep records of the following data on each person presenting with suicidal behaviour:
    - Demographic information
    - Average number of contacts made per individual
    - Whether a person was admitted to hospital or inpatient care

- Whether the individual accessed referral services or other services
- Consumer satisfaction on a 1-5 scale

100. The member of staff responsible for the systematic collection of data in the acute setting should record the following:

- Number of people treated after a suicide attempt in the last year
- Proportion of re-presentations by people with suicidal behaviour
- Proportion of people admitted
- Proportion of people receiving aftercare and follow-up
- The proportion of people entering the acute setting and discharged within 4 hours
- The proportion of people discharged from the acute setting who return within 28 days
- Proportion of people presenting who have made a suicide attempt, are experiencing thoughts of suicide who are provided with a Comprehensive Psychosocial Assessment
- Waiting times for a Comprehensive Psychosocial Assessment
- Proportion of people who are provided with a referral to an appropriate aftercare service.
- Proportion of people for whom an appointment is made with an appropriate ongoing care agency
- Proportion of people who receive a follow-up referral and appointment within 24 hours following discharge.
