# Supplementary material for: Development of best practice guidelines for suicide-related crisis response and aftercare in the emergency department or other acute settings: a Delphi expert consensus study
Source: BMC Psychiatry. 2019 Jan 7;19:6. doi: 10.1186/s12888-018-1995-1 (PMC6323675; doi:10.1186/s12888-018-1995-1)
Supplement: Supplementary file 3 — Guidelines for integrated suicide-related crisis and follow-up care in Emergency Departments and other acute settings. (PDF 2950 kb) [file 12888_2018_1995_MOESM3_ESM.pdf]

Guidelines for integrated suicide-related crisis and follow-up care in Emergency Departments and other acute settings

Published November 2017

LifeSpan Integrated Suicide Prevention

Black Dog Institute

Creating a mentally healthier world.

Acknowledgement
LifeSpan has been made possible thanks to the generous support of the Paul Ramsay Foundation. Our thanks go to those who participated in the research which led to the development of the present guidelines. We hope this report honours your contribution. We would also like to thank the team at the Black Dog Institute who contributed to this document: Nicole Hill, Lyndal Halliday, Nicola Reavley, Fiona Shand, Laura Vogl, Jo Riley, Isabel Zbukvic, and all the behind the scenes staff.

Suggested Citation
Hill, N.T.M, Halliday, L, Reavley, N.J (2017). Guidelines for integrated suicide-related crisis and follow-up care in Emergency Departments and other acute settings. Sydney, Black Dog Institute.

Contents

Message from the Director 5
Overview 6
Detailed Guidelines
- Description 12
- Scope of the guidelines 12
- Administrative and managerial roles and actions 13
- Resources needed for patients, carers, families, and clinicians 16
- Clinician and Suicide Response Team Roles and Actions 16
- Referral and follow-up arrangements 19

Appendix
- Clinical Summary

The
Black Dog Institute
is recognised as a global pioneer in the identification, prevention and treatment of mental illness, and the promotion of well-being. As a Medical Research Institute, clinical service and not-for-profit, Black Dog's focus is on rapidly implementing new scientific discoveries into real-world settings. The voice of lived experience informs everything we do, from our research through to our community outreach activities.

Black Dog is also spearheading Australia's largest suicide prevention trial – LifeSpan – which is setting best practice by taking a holistic approach that connects, amplifies and builds on existing suicide prevention services, tailored to each trial site region.

1. Kapur N, House A, Dodgson K, May C, Creed F. Effect of general hospital management on repeat episodes of deliberate self-harm: cohort study. BMJ. 2002;325:866-867.
2. Kapur N, Steeg S, Webb R, et al. Does clinical management improve outcomes following self-harm? Results from the multicentre study of self-harm in England. PLoS ONE. 2013;8 (8):e70434. doi:70410.71371/journal.pone.0070434.
3. Bostwick JM, Pabbati C, Geske JR, McKean AJ. Suicide attempt as a risk factor for completed suicide: Even more lethal than we knew. American Journal of Psychiatry. 2016;173(11):1094-1100.
4. Huisman A, Kerkhof JFM, Robben PBM. Suicides in users of mental health care services: Treatment characteristics and hindsight reflections. Suicide and Life-Threatening Behavior. 2011;41(1):41-49.

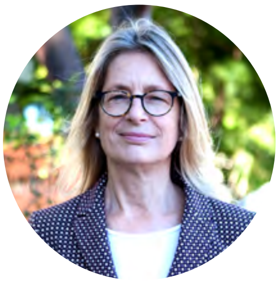

Message from the Director

Research shows that the quality of care someone receives from the Emergency Department (ED) following a suicide attempt can influence their risk of attempting or dying by suicide in the future.[1-4] This means that crisis and follow-up care from the ED can be life-changing for the person as well as their loved ones.

For many experiencing suicidal crisis, the ED is the best available option to obtain the essential safety and support they need at that time. It may also be the person's first point of contact with professional help. This is true whether they are referred by a health professional, emergency service, or loved one.

At the Black Dog Institute we have heard a clear call from those with lived experience of suicide that the care many receive in the ED inadequately addresses their needs. We have also heard from clinicians that suicide risk assessment, the current focus of emergency department care in many locations, offers insufficient guidance to inform treatment and support planning. The development of new best practice guidelines aims to address these concerns and more, providing a new benchmark for the health system and ensuring a life-affirming experience for every person who presents to the emergency department in suicidal crisis.

Scientia Professor Helen Christensen

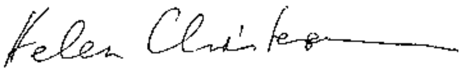

Director, Black Dog Institute

Overview

The Guidelines for Integrated Suicide-Related Crisis and Follow-Up Care in Emergency Departments and Other Acute Settings have been developed with the input of health care professionals and people with lived experience of suicide. It is hoped that these guidelines can be used by those working in acute settings to inform service planning, better equip and support staff to work effectively with those at risk of suicide, and guide empathic, compassionate responses to people experiencing suicidal crisis.

Patient and carer experience in the Emergency Department

Despite evidence that quality of care in the Emergency Department (ED) can influence the likelihood of future suicide attempts, a recent comprehensive Australian review of care after a suicide attempt found very low levels of satisfaction with ED care[5]. Patients and carers reported that their emotional distress was not attended to; many believed they were discharged too rapidly and were left to seek their own options for ongoing care. To ensure that every person leaves ED feeling safe and supported, it is vital that EDs are equipped to care for people in suicidal crisis[5].

Challenges in the Emergency Department

An environment characterised by constant demand, the ED poses unique challenges for providing care in a suicidal crisis. For clinical staff, it can be challenging to adequately address complex needs within strict organisational and legal boundaries. Very public consult settings and short triage times can make building patient-clinician rapport difficult, for instance, and Australian ED clinicians report a lack of confidence in assessing and managing suicide related presentations[6]. Ensuring processes are consistent with best practice can also be difficult when general resources are stretched, such as staff availability at certain hours or across geographic areas. An essential focus on managing acute presentations often means that ED care doesn't integrate smoothly with community-based care pathways.

Unfortunately, policy and system guidelines on ED care often state *what* needs to be done, but without adequate detail on *how* to do it. To be effective, recommendations for evidence-based care must first be feasible within the unique context of acute care settings. They must consider the experiences of those who manage and provide ED care, as well the experiences of patients and their family or carers.

Setting the benchmark with best practice Guidelines

Guidelines for Integrated Suicide-Related Crisis and Follow-Up Care in Emergency Departments and Other Acute Settings provide recommendations based on the research-evidence for best practice care for suicidality, while addressing the issues that have led to low patient and carer satisfaction with ED care. Developed as part of the LifeSpan systems approach to suicide prevention,

5. NHMRC Centre of Research Excellence in Suicide Prevention (C.R.E.S.P). Care after a suicide attempt. 2015.
6. Jelinek GA, Weiland TJ, Mackinlay C, Gerdtz M, Hill N. Knowledge and confidence of Australian emergency department clinicians in managing patients with mental health-related presentations: findings from a national qualitative study. International Journal of Emergency Medicine. 2013;6:2-2.

the guidelines represent a core component of the strategy aimed at *Improving Emergency and Follow-Up Care for Suicidal Crisis* (see page 9 to find out more about LifeSpan).

These Guidelines build on previously published recommendations and jurisdictional guidelines, while also including the perspectives of people with a 'lived experience' of suicidal crisis in an ED setting. This experience might be as a patient or consumer or a family member, support person or carer. Including this perspective provides vital insight into what is most valuable for recovery, and is the reason that *Lived Experience Inclusion at Every Level* is a core component of the LifeSpan approach to suicide prevention. These recommendations and lived experience perspectives have been combined with insights from clinicians reflecting what is most critical, practical and feasible in the ED.

All activities in LifeSpan aim to build on the existing evidence-base in suicide prevention by incorporating key principles from knowledge translation and implementation science. The Guidelines provide a full picture of requirements for quality care including information for administrators and managerial staff as well as a *Clinical Summary* focused on practical steps to be implemented in real time. By taking the guess-work out of how to create change on the ground, this guide will make it easier for administrators to determine appropriate resourcing, as well as for clinicians to provide the highest quality care.

Implementation of the Guidelines will fulfil requirements under NSW Health Policy Directive for Clinical Care of People Who May Be Suicidal (Document number PD2016_007).

The research behind the Guidelines

The Guidelines were produced using the 'Delphi' method, a world standard for developing empirically-based expert recommendations. This method involves asking panels of experts to rank items in terms of importance, until the highest level of consensus is reached.

In this case, items were included if they related to care for suicidality in an acute setting or following a suicide attempt. Items were collected by systematically searching international literature (including websites, reports and journal articles). This resulted in a collection of recommendations from guidelines developed in the UK, Canada, and Australia, which were added to statements from people with experience working in crisis settings or aftercare services, as well as people with a personal experience of suicide. Only those items considered significant by all panel members were included in the final guidelines. This resulted in a core set of best practice Guidelines from over 400 original items.

A total of 89 individuals participated on the panel, including 50 people with lived experience and 39 professionals representing emergency department staff or employees in acute settings, health care providers, employees of a crisis response or aftercare service, academics and people employed in the non-profit community outreach sector.

The full Delphi study will be published following peer review.

This accompanying version of the Guidelines including the Clinical Summary is being disseminated separately, to provide practical and realistic strategies for translation into clinical practice and for use within the LifeSpan research trial.

Beyond the Emergency Department

For many people, receiving care in the acute setting is the first step on their journey to recovery. Research shows that the transition from ED to aftercare following a suicide attempt can have powerful effects on someone's future risk of self-harm[7]. For this reason, access to high quality aftercare represents another essential element in any suicide prevention initiative.

The Guidelines address the steps to be taken by Emergency Department staff to arrange, in conjunction with the patient and (with the person presenting in suicidal crisis's consent) their family or support people, for referral and follow-up prior to discharge.

As part of the LifeSpan trial, a separate set of evidence-based guidelines for aftercare services have been prepared.

Our next steps

The impact of these Guidelines and Clinical Summary will be evaluated through the LifeSpan Cohort Study. The aim of this study is to measure whether the experience of people at risk of suicide, family/carers, as well as health care staff (who have contact with people at risk of suicide) changes over the LifeSpan trial. The study also aims to compare these experiences over time with those of people from non-trial sites, where LifeSpan has not been implemented. Data will be gathered from interviews, as well as through routinely collected data. By linking key datasets, the study will follow patients through outpatient care as well as any re-presentations to ED or re-admissions to hospital.

The outcomes of this evaluation will be published following peer-review, adding to the evidence-base relating to acute care for suicidality. Alongside this, Black Dog Institute will continue to advance towards best practice care for suicidal crisis in the emergency department, by working with health professionals, policy makers and people with lived experience of suicide.

7. Krysinska K, Batterham PJ, Tye M, et al. Best strategies for reducing the suicide rate in Australia. Australian and New Zealand Journal of Psychiatry. 2016;50(2):115-118.

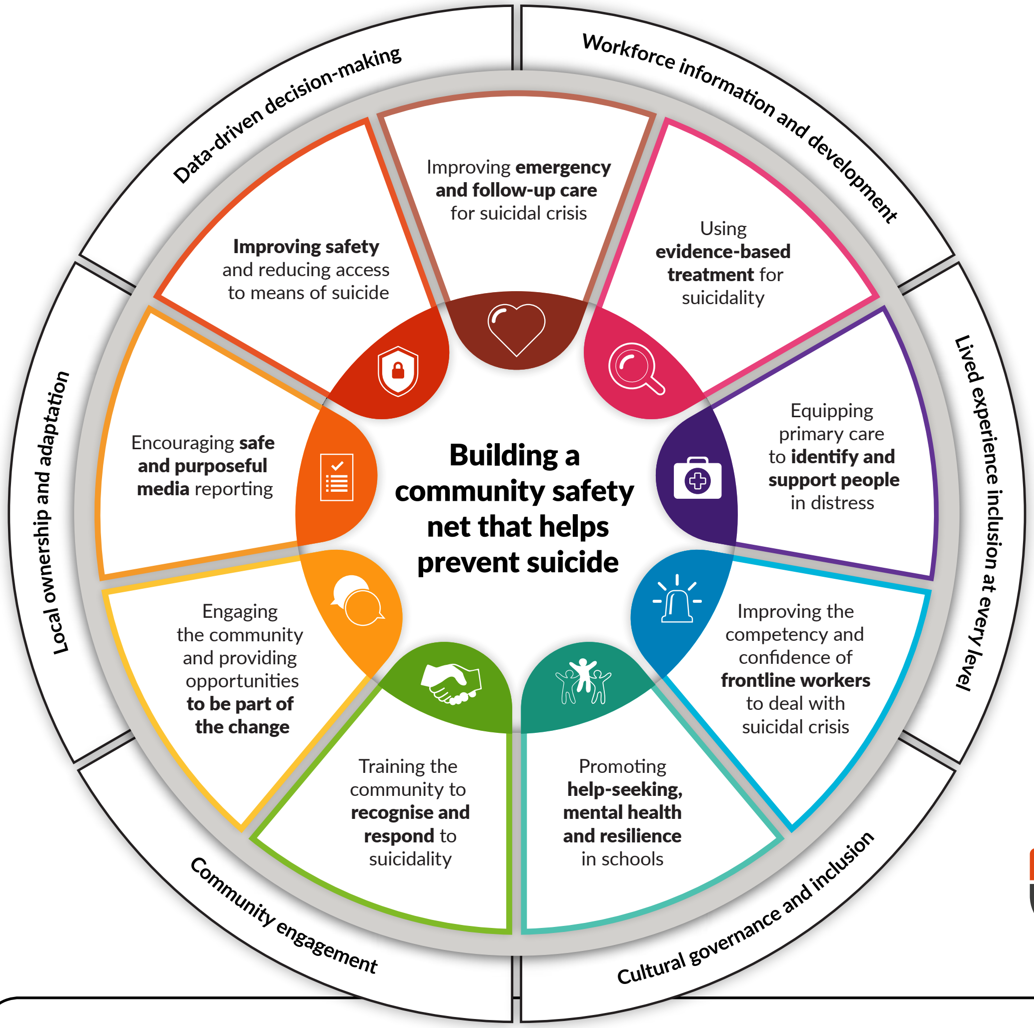

LifeSpan™ Integrated Suicide Prevention

LifeSpan is a new, evidence-based approach to integrated suicide prevention. It combines nine strategies that have strong evidence for suicide prevention into one community-led approach incorporating health, education, frontline services, business and the community. For each strategy, LifeSpan selects and implements the interventions or programs that have the strongest evidence-base. In doing so, LifeSpan aims to build a safety net for the community by connecting and coordinating new and existing interventions and programs, and building the capacity of the community to better support people facing a suicide crisis.

Estimates suggest that the simultaneous implementation of all LifeSpan strategies by well-connected Local Implementation Teams may be able to prevent up to 21 per cent of suicide deaths and 30 per cent of suicide attempts.

LifeSpan has been developed by the Black Dog Institute in partnership with clinicians, researchers, community groups and people with lived experience of suicide. It is funded by the Paul Ramsay Foundation and is being trialled in four regions in NSW – Newcastle, Illawarra Shoalhaven, Central Coast, and Murrumbidgee – using a stepped-wedge, randomised design. The local trials will run for an initial period of two and a half years (including a six-month planning period).

To find out more about LifeSpan visit www.lifespan.org.au

‘You can’t hear my silent screams and you can’t see all of the wounds inside my tortured body and my mind. Maybe, just maybe, you can see the hopelessness etched deep inside my eyes. The things you say to me and the way you react to me will be critical to my ability to live. Do not prejudge me for you may never know what drove me to this point in my life.

These words could apply to many who present at an Emergency Department after attempting to end their own life, or to those who are considering ending their own life. I know that is how I felt, and how many others whose lives have been affected by a suicide attempt or suicidal thoughts have felt. You can’t underestimate the importance of being treated with compassion and respect when seeking help during a suicidal crisis; it connects you back to life and hope.

ED staff play a key role in giving a person something they are desperate to find again – hope that they can recover and overcome the issues that drove them to this terrifying point in their lives. Hope helps create a plan for the care and support they need to move through their crisis and into recovery. Once the plan is in place, it’s critical to follow-up to make sure the person remains supported and stays on track with the plan. If done well, it’s like helping them to build a bridge back to life and offering to walk with them out of the darkest place they have ever known.’

Allan Sparkes CV, OAM, VA

Lived experience speaker,
author and advocate

‘Family or friends are usually the main source of support for a person in suicidal crisis and are desperate to do all they can to help, to keep their loved one safe. It’s an extremely challenging and frightening time but it helps to be equipped with relevant information and actively involved in support planning. These guidelines set the standard for ensuring everyone who presents at emergency, including their family or friends, are treated with dignity and respect, receive practical support and appropriate referrals to the care they need to recover.’

Bronwen Edwards

CEO, Roses in the Ocean,
and lived experience advisor

‘In recent years clinicians became fixated on “risk assessment” when trying to understand and assist people who raised concerns about suicide. Now that it is clear that suicide risk assessment offers no useful assistance in this task, many clinicians are asking for aid in helping the suicidal patient. These guidelines offer a comprehensive framework to approach patients with the aim of developing a good understanding of their predicament and negotiating with them and their families the best way to achieve the outcomes they want and live the lives they strive for.’

Dr Christopher Ryan

Clinical Associate Professor and Consultation-Liaison
Psychiatrist, University of Sydney and Westmead Hospital

‘Our research indicates some clear opportunities for improving the way in which we respond to a person in suicidal crisis. From our interviews with people who had experienced the health care system following a suicide attempt, the message was clear: an empathic, compassionate response to a person in distress is not an add-on to treatment, it is intrinsic to the treatment. There is a clear association between patients’ satisfaction with the first response they receive from health services and their willingness to seek further help. Our hospital staff need to be supported to work effectively with at-risk patients in order to reduce the risk of further suicide attempts.’

Dr Fiona Shand

Senior Research Fellow and LifeSpan Research
Director, Black Dog Institute

LifeSpan Integrated Suicide Prevention | Black Dog Institute

Guidelines for integrated suicide-related crisis and follow-up care in Emergency Departments and other acute settings

1 Description

These guidelines outline the team structure, skills, and actions that staff can take when responding to a suicidal person in acute settings such as hospital Emergency Departments and Psychiatric Emergency Care Centres. The guidelines were developed using the Delphi consensus method. Two expert panels, one consisting of health care professionals and the other consisting of people with lived experience of suicide, rated each action according to how important they believed it to be for inclusion in the guidelines. Only those items that were endorsed as essential or important by at least 80% of both panels were included in the final guidelines. It is hoped that these guidelines can be used by those working in acute settings to guide the response to people who have made a suicide attempt or are experiencing thoughts of suicide.

2 Scope of the guidelines

While there are a number of models for responding to suicidal crises, which vary between countries and states, the focus of these guidelines is on promoting best practice within existing service models rather than proposing an entirely new model. Therefore, in these guidelines, we use the term The Suicide Response Team to refer to members of the hospital Mental Health Team who are trained in suicide prevention and responsible for the coordinated response to suicide presentations. The team may be comprised of multiple members with designated roles, or a single clinician, such as a mental health nurse who is trained to perform multiple roles in response to suicide presentations. In these guidelines, the term 'a person at risk of suicide' is used to refer to a person who has made an attempt or is having thoughts of suicide.

3 Administrative and managerial roles and actions

This section covers areas of work for which administrators are responsible, such as staffing, resourcing, contracting arrangements, data collection and systems, and room allocation arrangements. These responsibilities may be split across hospital administrator/s and clinical administrator/s.

3.1 Staffing

The manager responsible for staffing should ensure that the SRT is staffed 24 hours a day or that caseworkers are available after-hours to respond to crises. They should accommodate for known high-demand periods by increasing the number of staff who have the skills and experience to assess and treat people at risk of suicide. In order to understand when rates of people at risk of suicide presenting to the acute setting are highest, a designated member of staff in the acute setting should be responsible for collecting the following data:

a. Number of presentations
b. Time of presentation
c. Day of presentation
d. Number of people who did not wait for treatment
e. Time waiting for consultation by the suicide response team (SRT).

3.1.1 Staffing the acute setting without an SRT

The member of staff responsible for staff scheduling (also known as the staff roster) should ensure the hospital without an SRT is staffed by doing the following:

- Hiring additional providers who have the clinical skills and experience to work with people who are at risk of suicide
- Contracting agencies in the community who can provide services such as monitoring, assessment, referrals and follow-up
- Ensuring that an after-hours clinician is available to assess people at risk of suicide presenting to the acute setting outside regular hours
- Ensuring that a psychiatry register or psychiatry liaison is available on call, at all times
- Ensuring that an after-hours mental health nurse is available to assess people at risk of suicide presenting to the acute setting outside regular hours
- Ensuring that a person trained in comprehensive psychosocial assessment, supportive counselling and intervention is available 24/7
- Allocating providers from other departments in the hospital during periods of high demand.

3.2 Providing a safe environment

Staff in acute settings should ensure that the environment is safe by providing a comfortable place for the person to sit and wait for consultation or to complete assessment procedures. A signed and dated environmental safety audit should be kept on record in the acute setting. This should cover removal of ligature points and access to means of suicide.

3.3 Training

Nursing Directors and the clinical supervisors of the medical staff should be accountable for ensuring that all staff members who have contact with a person at risk of suicide are trained. The manager responsible for training and education in the acute setting should assess the training needs of staff and should ensure that training is provided to the following staff:

- All staff in the acute setting who have contact with people at risk of suicide
- Paramedics (including ambulance staff)
- Social workers
- Youth worker
- Acute setting security
- Other allied services that attend to person's in the acute setting.

In acute settings without an SRT, the responsible manager should ensure that staff receive more comprehensive training in suicide and mental health. The manager should ensure that staff members working with people with suicidal behaviour have the opportunity for:

- Supervision
- Peer discussion and support
- Avenues to discuss and understand their own reactions
- Case review meetings and opportunity for supervision
- Access to appropriate clinical supervision, consultation or advice from a senior clinician at all times.

3.3.1 Core competencies

The following is a list of core competencies that SRT members should demonstrate:

3.3.1.1 Knowledge

- Hospital policies, protocols and guidelines
- Basic understanding of medico-legal issues in the delivery of mental healthcare as well as requirements of common law and legislation
- Knowledge of the different presentations of suicidal behaviour in different age groups and identifying stressors that lead people to suicide
- Knowledge and recognition of comorbidities and their impact on suicidal behaviour (e.g. alcohol and drug disorders), including brief interventions and detoxification programs
- Sequence of care in the acute settings
- Mental capacity and its application in the acute setting
- Procedures for engaging partnering services who have shared governance of the person's care
- Detailed understanding of all local, evidence based, resources relevant to the support of service users
- The impact of cultural differences on influencing symptomatology, perception of symptoms, help seeking behaviour, and clinical judgment
- Procedures to notify police of firearms or weapons present in the home
- Procedures for contacting child protection services.

3.3.1.2 Skills

- Ability to perform detailed evaluations of suicidal behaviour and management of self-harm
- Ability to perform mental state assessments and comprehensive psychosocial assessments
- Problem solving techniques
- Recognises and responds to frequent attenders
- Recognises and responds to acute behavioural disturbances
- Managing treatment adherence
- Mental health triage
- Ability to perform culturally relevant and sensitive assessments
- Ability to generate and implement management/ care plans
- Ability to assess hostile or guarded people
- Ability to formally assess a person's decision-making capacity when they disagree with treatment recommendations or if they decide to leave the acute setting before completion of the assessment
- Ability to inform a person of their treatment options, particularly if they have limited judgment
- Use of corroborative information to aid diagnosis, assessment, management, and discharge planning
- Ability to provide means restriction counselling which informs the person's carer, family or friends about the dangers of access to medication, and other lethal objects.

3.3.1.3 Skills in person-centred care

- Ability to engage the person as partner in the design of their care
- Ability to listen and talk to the person, explaining actions, and providing reassurance
- Person-centred engagement, reflective practice and building a therapeutic alliance
- Responding respectfully, in a non-stigmatising, non-discriminatory manner
- Understanding of the impact of emotions and feelings on interactions with others, including regulating emotions and feelings
- Understanding of the impact of attitudes and judgments on help-seeking behaviour
- Acting in ways to make people feel validated and listened to
- Offering comfort, reassurance and hope to the person.

3.4 Facilitating collaboration

The manager responsible for facilitating collaboration and coordination across health and social care services should ensure that the following are in place:

- A standardised form for communicating with partnering community services
- Healthcare information exchanges to exchange records and coordinate services with community partners
- Joint education opportunities such as coordination of inter-professional education programs
- Arrangements for different providers to present at formal professional development events
- Joint staffing meetings with inpatient units and acute psychiatric care departments
- Mentoring
- Use of telepsychiatry or videoconference (e.g. Skype) to close provider gaps if local resources are limited
- Provision of training in risk management

- Facilitation of feedback and communication streams for service issues
- Allocation of time in the acute setting for designated mental health providers from other hospital departments to assess and treat a person presenting for suicide behaviour
- Provision of efficient and standardised data collection across sectors
- Provision of routine screening for dual diagnosis across sectors
- Provision of adequate space and resources (rooms, computers) for co-located services.

3.5 Service evaluation

A member of staff in the acute setting should be responsible for implementing a mental health data collection and monitoring framework that assesses service activity and quality. This should include a process for asking people with lived experience for feedback on their service experience.

The SRT should keep records of the following data on each person presenting with suicidal behaviour:

- Average number of contacts made per individual
- Whether a person was admitted to hospital or inpatient care
- Whether the individual accessed referral services or other services
- Demographic information (including indicators of LGBTI, Aboriginal and Torres Strait Island status)
- Whether the person attended the acute settings alone
- Whether the person required a translator.

The member of staff responsible for the systematic collection of data in the acute setting should record the numbers or proportions of people who:

- Are treated after a suicide attempt (in the past year)
- Re-present with suicidal behaviour
- Are admitted
- Receive aftercare and follow-up
- Enter the acute setting and discharged within 4 hours
- Are discharged from the acute setting who return within 28 days
- Have made a suicide attempt, are experiencing thoughts of suicide and who are provided with a Comprehensive Psychosocial Assessment. Waiting times for a comprehensive psychosocial assessment should also be recorded
- Are provided with a referral to an appropriate aftercare service
- Receive an appointment is made with an appropriate ongoing care agency
- Receive a follow-up referral and appointment within 24 hours following discharge.
- Receive a DCP
- Leave the acute care setting against the advice of the clinical team.

4 Resources needed for patients, carers, families and clinicians

Staff should ensure that written information on the following is available to those waiting for consultation and their family and friends:

- Referral and aftercare services available to the person through the hospital and/or services available in the community
- Alternatives to inpatient admission (e.g. community crisis houses, day hospitals, home treatment, respite)
- Community support groups and other relevant local community programs
- A person's rights in healthcare
- Available mental health crisis services (e.g. mental health drop in clinics)
- Any non-clinical services that may alleviate isolation and promote hope.

4.1 Community referral resource manual

The SRT should develop and maintain a resource manual of local outpatient mental health providers and community services to assist with referral, follow-up and continuity of care. This should include:

- A description of the service provided
- A description of eligibility criteria
- Relevant skills of the agency/service
- Specialisations of the service provided
- Contact information
- Referral information

5 Clinician and Suicide Response Team Roles and Actions

5.1 Team roles

The suicide response team (SRT) should have members co-located in the department where people at risk of suicide receive acute care. The member of staff who is responsible for overseeing the operations of the mental health team should ensure that all SRT members receive appropriate training in the care of a person at risk of suicide. The SRT should include people trained in liaison psychiatry; delivery of psychosocial assessments; crisis intervention; the provision of follow-up care; discharge, formulation of the discharge care plans and transfer of care; and internal hospital referrals and referrals to external services. If the SRT liaison psychiatrist is in training, they should have 24/7 access to a consultant psychiatrist. SRT members should:

- Ask the person whether they prefer to wait in a private or public place and should ensure that the person is not left unsupervised
- Have a protocol for a person that presents to the acute setting alone
- Notify key hospital workers or case-managers previously involved with the person's care
- Link the person to hospital services that address comorbidities (e.g. drug and alcohol services)
- Refer the person to inpatient care when necessary (e.g., in cases of people with command hallucinations or ready access to lethal means)
- Follow-up people transferred to different hospital wards (e.g. following surgical intervention) to ensure they receive a comprehensive psychosocial assessment
- Provide emotional support and help to carers, family or friends accompanying the person and who are experiencing high levels of stress and anxiety
- Provide carers, family or friends with information about caring for someone with suicidality
- Have a protocol for contacting the person's GP in order to assist in identifying any relevant medical history
- Provide referrals for carers to local services and support groups
- Identify people who frequently attend the acute setting
- Arrange the comprehensive psychosocial assessment
- Complete discharge care planning and refer the person to follow-up with aftercare services.

5.2 Acute settings without a SRT

Some acute settings may not have a fully staffed mental health team. In this instance, the member of staff who is responsible for coordinating the mental health team should arrange for a 24-hour mental health telephone triage service that offers instructions and advice to people

prior to presenting to the acute setting. They should also arrange for partnerships with allied health professionals, ambulatory care services, community-based services (e.g., local NGO's, outpatient services, and social services) and services that provide cultural support services for Aboriginal and Torres Strait Islander people. There should also be links with a community-based team to assist with aftercare and follow-up.

5.3 Initial contact

Initial contact is the first point of contact with medical services made by a person at risk of suicide, a person's carer, or clinical practitioner. A person who has received the required medical treatment should be triaged directly to the SRT. Staff in acute settings should offer to contact a family member or friend of the person.

5.4 Provision of peer support

Acute settings with peer support workers should have supervision and governance structures in place to support them and should arrange for them to receive appropriate training for response in the acute settings.

5.4.1 The role of the peer support worker

The peer support worker should assist the person at risk of suicide in the following ways:

- Advocating for the person to be treated with dignity and respect
- Offering to provide basic physical comforts such as blankets, towels, meal trays, reading materials, and water
- Offering to accompany the person while they wait for consultation with the SRT
- Offering to help the person communicate with staff in the acute setting
- Offering to liaise with the person's carer, family or friends

5.5 The Comprehensive Psychosocial Assessment

Comprehensive psychosocial assessment refers to the evaluation of a person's mental, physical and emotional health, as well as their ability to function in the community. This assessment plays a central role in helping to improve the aspects of the person's life that have contributed to their risk of suicide and offers an opportunity to show compassion and understanding. A SRT member should administer a comprehensive psychosocial assessment to everyone who is referred for a suicide attempt or thoughts of suicide and should assess the needs of carers accompanying the person at risk of suicide. If the SRT member cannot complete a comprehensive psychosocial assessment because the person refuses, the SRT liaison psychiatrist should assess the person's mental capacity.

The SRT should ask treating health professionals and people in the person's social network to provide information on their beliefs about the current presentation, behavioural changes in the person and access to means of suicide such as firearms or stockpiles of medication. They should also assess the needs of carers, family or friends accompanying the person.

5.5.1 Content of the Comprehensive Psychosocial Assessment

The comprehensive psychosocial assessment should include assessment of the following:

Suicidality

- Assessment of the physical injury, its severity and the potential lethality of the chosen method
- The person's subjective view about the lethality of their attempt
- The circumstances that led to the suicide attempt including suicidal ideation and persistence of suicidal ideation
- Assessment of current suicidality, including the motives underlying the suicide attempt
- Assessment of current/ongoing suicidality (thoughts, plan, lethality of plan, level of intent, access to means)
- Evidence of preparatory behaviours and planned precautions to prevent discovery or interference of the person's suicide attempt
- A comprehensive mental state examination
- Assessment of negative feelings including depression, hopelessness, helplessness, loneliness, feeling trapped, and continuing suicidal intent
- Ambivalence about living or dying
- Evidence of covert suicidal ideation (e.g., making a will, paying debts, hinting – "you will not have to worry about me anymore")

Medical and psychiatric history

- Suicide attempt and self-harm history
- A review of mental health symptoms

- A review of current physical health symptoms and diagnoses
- Medication and substance use history
- Ability to maintain sufficient hydration and nutrition

Psychosocial history and life stressors

- The person's support resources
- Whether the person has any dependents and parenting skills
- Exposure to domestic violence, neglect or abuse
- Ability to fulfill family and occupational responsibilities
- Exposure to someone else's suicidal behaviour or suicide death.

Presence of risk factors for suicide

- The circumstances that led to the suicide attempt including suicidal ideation and persistence of suicidal ideation
- The person's perceived burden on others
- Trauma history and treatment needs
- Veteran status and war trauma
- Whether the person comes from a cultural minority group (e.g. Aboriginal and Torres Strait Island people, LGBTI)
- Impulsiveness and risk taking behaviours and the person's acknowledgement of self-destructive behaviours, if applicable.

Ability to recover in the community

- Assessment of protective factors, e.g., family support, coping skills
- The person's ability to seek and access help and identify any barriers to accessing services including an assessment of financial barriers
- The ability of the person to enter into a therapeutic alliance/partnership including the persons engagement with help
- The person's positive coping and problem solving skills
- An assessment of family and social connectedness
- Ability to interact with others
- Identify the core values/beliefs, goals and strengths of the person
- Identify problem-solving strategies the person is open to
- The person's concerns about stigma
- Immediate medium and long term mental health needs
- Immediate and long term social need.

The psychological assessment should also include psychological first aid and problem-solving counseling that addresses the persons needs and is aligned with strategies that the person has identified and is open to.

5.5.2 Using the Comprehensive Psychosocial Assessment to guide treatment

The SRT should use the results from the comprehensive psychosocial assessment to complete the following:

- Develop a treatment care plan
- Provide education about the person's condition and treatment options
- Provide a referral for psychiatric consultation, if applicable.
- Provide referrals to other hospital or outpatient services such as mental health treatment, substance abuse treatment, family counselling and other social services etc.
- Educate carers, family or friends about the person's condition and treatment options

Where a clinical decision results in the person going home, the SRT should organise a follow-up assessment through relevant care coordination programs and should request the person's consent before sharing information from the Comprehensive Psychosocial Assessment.

5.6 Crisis management plans for frequent attenders to acute settings

People who frequently attend the acute setting for mental health related behaviours should be encouraged to develop a crisis management plan that sets out a person's preferences for future medical treatment. This should be done when the person is well and gives the person the opportunity to work with health professionals to decide the type of treatment they would like to receive in a time of crisis.

6 Referral and follow-up arrangements

Referral describes the transmission of a person's personal or health information between one agency and another for further assessment, care or treatment. Follow-up, which is also known as aftercare, describes the continuity of care following discharge from acute settings.

6.1 Arranging referral

The SRT should arrange referral and follow-up services based on the needs identified by the comprehensive psychosocial assessment. The SRT is responsible for the following:

- Confirming that the person's referral has been received by the relevant aftercare service
- Arranging a follow-up referral to the person's GP
- Documenting referral outcomes
- Arranging referrals to local services and support groups for carers, family or friends and to postvention and bereavement services for carers, family or friends of a person who died by suicide after entering the acute setting.

The SRT should contact relevant agencies prior to making a referral to ensure the agency can accommodate the timely implementation of the referral request. They should ensure that aftercare referrals are established within 24- 72 hours for all cases. However, referrals to services involving the person's immediate needs should be prioritised as urgent meaning referral cannot wait and must occur the next day.

6.2 Arranging follow-up

The SRT should arrange follow-up services for everyone who has presented to the acute setting having made a suicide attempt or experiencing thoughts of suicide. Follow up support should be negotiated with the client to ensure they get the care and support needed without breaking confidentiality and may be done face-to-face or by using 'Crisis cards' with emergency phone numbers and safety measures. The SRT member responsible for follow-up should have access to the patient administration systems that contain information about people requiring follow-up. Rural and remote communities should partner with other organisations (e.g. Red Cross, Lifeline, police, etc) to provide follow-up contact via phone, Skype, email or home visits.

The SRT should have a protocol to assist in managing the care of a person who does not give consent for communication with aftercare and follow-up services. Staff should be trained in understanding the difference between requesting and requiring consent and the importance of mitigating harm.

6.3 Discharge and transfer of care

6.3.1 The discharge care plan

The purpose of the discharge care plan (DCP) is to ensure a safe and successful transition for the person from the acute setting to the community. It provides a means of synthesising assessment information and agreed strategies and is particularly important for people with multiple and complex needs. It specifies the steps that must be taken and assists the person to come to a decision that is appropriate for their needs, wishes, values and circumstances.

The DCP should contain strategies that are accessible, available and valued by the person, their family and/or care. It should be written using non-stigmatising language (e.g. avoid the use of terms such as 'non-compliance') and should include culturally appropriate assessments and referrals, as well as developmentally appropriate language. The DCP should include the following:

Treatment and support recommendations

- Recommendations and actions that address needs identified in the comprehensive psychosocial assessment
- Instructions for the person's medication including frequency, dosage and side effects
- A schedule of appointments for follow-up and aftercare including contact details of all the aftercare services included in the person's referral
- Contingency arrangements for contacting specialists
- Arrangements to overcome barriers to accessing aftercare services
- Housing support recommendations and action needed to secure accommodation (if applicable)
- Recommendations to reduce social isolation and engage with a support network
- Relapse prevention recommendations
- Specific steps to seek help and support if symptoms re-occur or worsen or the situation deteriorates following discharge
- Follow-up procedures following non-compliance or failure to attend aftercare appointments
- Education about warning signs of possible relapse and what to do
- Strategies to mitigate intolerable distress, pain, coping, and suicidal thoughts at home
- Harm minimisation plan for alcohol and drug use (if applicable)
- Emergency contact details
- Details of the person's nominated support network and related contact details
- 24/7 mental health emergency contact details (including phone support services such as Lifeline)
- Contact details for crisis assistance and community mental health services.

6.3.2 Providing the DCP to the person

The SRT should provide a DCP to everyone who presents to an acute setting for suicide risk. Team members should work with the person to develop the DCP.

The SRT should provide the DCP to the person before they are discharged or transferred from the acute setting, although they may provide a brief DCP to the person when arrangements have been made to develop the care plan in the community during a scheduled appointment. In such cases, the SRT should make arrangements to ensure all people who receive a brief DCP are provided

a more extensive DCP within 24 hours of discharge. The SRT should engage in more extensive discharge planning for people with multiple needs identified by the psychosocial assessment.

The DCP should be provided to the person in written format, verbally, and in any other preferred delivery determined by the person (email, mail). The SRT should also provide a written copy of the DCP to the person's GP or aftercare service within 24 hours of the discharge, and, with the person's consent, to their carer, family or friends.

6.3.3 Discharge eligibility

For a person eligible for discharge, the SRT ensures that:

- The contact details of the person, their GP, as well as those supporting them in the community, are updated in the hospitals electronic records
- The person has received the following:
  - The DCP
  - Medication and a prescription supply, if applicable
  - Information stating the benefits of follow up and treatment adherence
  - Information such as crisis cards, business cards, and brochures from community services and partners
- The person has adequate arrangements to be transported to a safe location at discharge
- Phone hand-over to the relevant agency (during regular business hours) has been performed.

6.3.4 Transfer of care

If appropriate and with the person's consent, the SRT should assist with finding a substitute treatment setting, including inpatient, sub-acute or private hospital beds, respite care or special accommodation. In such cases, the SRT is responsible for handover and for providing all acute setting notes and a copy of investigation results to the staff in the new setting.

During discharge, the SRT should carry out the following procedures:

- Notification of the person's GP
- Notification of community-based services used by the person (with the person's consent)
- Arranging support for dependents and carers, family or friends
- Exchanging information between service providers (with the person's consent)
- Arranging reviews and reassessments.

If possible, the SRT ensures communication with services involved in the person's aftercare is made before the person is discharged.

6.3.5 Discharge summary

The SRT provides a discharge summary that includes:

- The person's condition/diagnosis/crisis
- Reason for attending the acute setting
- Summary of the assessments performed
- Interventions undertaken by the SRT
- Summary of the DCP
- Interventions arranged
- Recommendations to the person's GP if the person needs additional help

The SRT provides the discharge summary to the following people:

- The person
- The person's GP
- The person/service responsible for follow-up
- All services involved with the person's aftercare (including follow-up and outpatient services)

The SRT should provide the discharge summary within 24 hours of discharge. The person can request the discharge summary is provided in a format best suited to them (e.g. Email)

6.3.6 Provision of the discharge summary to carers, family or friends

The SRT should ensure that the person's carer, family or friends are provided with discharge information with the person's consent and should document whether the person has given this consent. Information given to carers, family or friends includes:

- Safety measure advice including means restriction counselling (i.e. securing medications)
- Harm minimisation techniques
- Healthy strategies to help with person cope with distress
- Encouragement to support the person being discharged
- Specific risks related to the person
- Crisis and emergency contact numbers
- Explicit contingency arrangements so the carer can contact specialist services if they need to
- Advice about how to handle situations in which the person is unwell but avoiding or resisting help.

6.3.7 Case management

Follow-up care and case management may be provided by the SRT or by a community-based service. In the latter case, the SRT should liaise with service providers to ensure referred aftercare has been received. Effective case management involves:

- Expressing concern and support to the person and explaining the purpose of the follow-up contact
- Discussing the person's discharge care plan, checking their progress and assess whether the discharge care plan has been useful
- Facilitating engagement with relevant services and providing additional referrals to community supports, when applicable
- Determining whether treatment has been sought, organised, and delivered
- Reviewing barriers to treatment adherence and developing alternative strategies which encourage the person to return to treatment
- Providing telephone reminders of appointments
- Assessing the success of the person's transition back into the community and discussing future case management planning
- Establishing a more suitable therapeutic plan in collaboration with the person and health services (if applicable)
- Conducting a mood check
- Discussing solutions to problems and the management of stressors
- Explaining when the schedule of follow-up contact will end
- Inviting the person to stay in touch and call whenever they feel they are in crisis.

First follow-up contact should occur within 7 days.

6.3.8 Assertive follow-up

Some people may require assertive follow-up when discharged into the community. This includes people:

- With a suspected or diagnosed mental illness
- At risk of becoming homeless
- In situations of domestic violence
- Who cannot be sure they can keep themselves safe

- Presenting for suicide risk for the first time
- With a reported history of poor treatment adherence

Assertive follow-up should involve more frequent contact with the person, home-visits, intensive case management and out-reach support. First follow-up contact should occur within 24 hours for people requiring assertive follow-up.

6.3.9 Linkage with community services and aftercare

Community mental health services include urgent community-based assessment and short-term treatment interventions for people with mental illness in crisis, intensive long-term support for people with prolonged and severe mental illness and associated high-level disability, and non-urgent continuing care services for people with mental illness, and their carers, family or friends.

Inter-agency linkage

The SRT should arrange for inter-agency protocols that link the acute setting to community agencies, NGOs and community services. The protocol should cover:

- Aftercare referral arrangements
- Follow-up arrangements
- Assessment arrangements
- Treatment arrangements
- Shared care arrangements
- Agreement to allow some people to receive priority treatment when referred from the acute setting based on clinical need
- Access to clinical mediation and advocacy (e.g., social workers in aged care that can assist the individual with deciding treatment options)
- Outreach arrangements for hospitals that lack the capacity to employ in-house services
- Collaboration with GPs
- The responsibilities of each aftercare service when the person has multiple aftercare needs
- An agreement for accessing secondary consultation on request
- Agreement between community agencies that people with certain comorbidities (e.g. alcohol misuse) will not be excluded from aftercare services
- Information sharing arrangements.

LifeSpan Integrated Suicide Prevention

Black Dog Institute

Creating a mentally healthier world.

Guidelines for integrated suicide-related crisis and follow-up care in Emergency Departments and other acute settings

CLINICAL SUMMARY

Person presenting in suicidal crisis

Triage/refer to Suicide Response Team or Mental Health Team

- Provide safe comfortable environment with appropriate supervision/monitoring
- Offer to contact family member/friend
- Follow-up patients in different wards
- Provide peer support worker if available *(see reverse for role)*

Referral options and collateral information:

- Notify key hospital workers with prior involvement

Comprehensive Psychosocial Assessment

See reverse for full details

Treatment care plan - inpatient/outpatient

- Develop a treatment care plan
- Provide education about person's condition and treatment options

Arrange referral and follow-up services

In collaboration with person:

- for every patient going home, organise follow-up assessment and obtain consent before sharing information
- confirm referral has been received by relevant aftercare service
- confirm all referrals can be established within 24-72 hours of discharge and urgent cases the next day
- follow-up can include face-to-face, crisis cards etc.
- arrange a follow-up referral to the person's GP
- document referral outcomes

Referral options and collateral information:

- Refer to inpatient care when necessary
- Link to hospital services that address comorbidities (e.g. drug and alcohol)
- Referral for psychiatric consultation (if appropriate)
- Referral to other hospital/ outpatient services (e.g. Mental health treatment, substance abuse treatment, family counselling, social services)

Family/carer:

- Provide emotional support and help to carers/family/friends
- Provide information about caring for someone with suicidality
- Provide referral options for carers to local services/support groups

- Educate carers, family/friends about the persons condition and treatment options *(with the person presenting in suicidal crisis's consent)*
- Arrange referral to local services and support groups for family/friends/carers
- Referral to postvention/ bereavement services for family/friends and carers for a person who has died by suicide

Discharge Care Plan

Aim: To ensure safe successful transition from acute setting to community. Discharge Care Plan (DCP) should include: treatment and support recommendations, relapse prevention recommendations, emergency contact details *(see reverse for detail)*

Discharge eligibility:

- Update hospital records (details of person, GP, those supporting in community)
- The person has: DCP, medication and prescriptions, information on benefits of follow-up and treatment adherence, crisis cards, business cards, brochures of community services
- Arrangements to be transported to safe location at discharge
- Phone hand-over to relevant agency has been performed

Follow-up care/case management

- Express concern and support/explain purpose of follow-up contact
- Check progress and usefulness of discharge plan and update as required
- Facilitate engagement with relevant services/provide additional referral to community supports if applicable
- Determine whether treatment has been sought, organised and delivered
- Review barriers to adherence and problem-solve strategies to encourage return to treatment
- Provide telephone reminders of appointments
- Assertive follow-up required *(see reverse for priority populations)*

Family/carer:

Discharge information given to carers/family/friends should include:

- Safety measure advice - means restriction counselling
- Harm minimisation techniques
- Healthy strategies to help person cope with distress
- Encouragement to support person being discharged
- Specific risks related to person
- Crisis and emergency contact numbers
- Explicit contingency arrangements to contact specialist services
- Advice on how to handle the situation if the person is unwell and resisting/avoiding help

LifeSpan Integrated Suicide Prevention | Black Dog Institute

Creating a mentally healthier world.

CLINICAL SUMMARY

FURTHER DETAILS

Comprehensive Psychosocial Assessment (CPA)

The CPA is a broad assessment of a person's mental, physical and emotional health, as well as their ability to function in the community.

It should include the different domains below, but does not need to be carried out in a prescribed order.

IMPORTANTLY, the CPA is an opportunity to build rapport and show compassion and understanding.

Assessment of suicidality

- Assessment of the physical injury, its severity and the potential lethality of the chosen method
- The person's subjective view about the lethality of their attempt
- The circumstances that led to the suicide attempt including suicidal ideation and persistence of suicidal ideation
- Evidence of preparatory behaviours and planned precautions to prevent discovery or interference of the person's suicide attempt
- Ambivalence about living or dying
- Evidence of covert suicidal ideation (e.g. making a will, paying debts, hinting -'you will not have to worry about me anymore')
- Suicide attempt and self-harm history
- Impulsiveness and risk taking behaviours and the person's acknowledgment of self-destructive behaviours, if applicable
- The perceived burden on others
- Assessment of negative feelings including depression, hopelessness, helplessness, loneliness, feeling trapped, and continuing suicidal intent
- Assessment of current suicidality, including the motives underlying the suicide attempt
- Current/Ongoing suicidality (thoughts, plan, lethality of plan, level of intent, access to means).

Medical/mental health assessment and history

- A comprehensive mental state examination
- A review of mental health symptoms
- A review of current physical health symptoms and diagnoses
- Medication and substance use history
- Ability to maintain sufficient hydration and nutrition
- Psychosocial history and life stressors
- Current life stressors (e.g. financial, social, familial, occupational etc.)
- Whether the person has any dependents and parenting skills
- Ability to fulfil family and occupational responsibilities
- Trauma history and treatment needs
- Exposure to domestic violence, neglect or abuse
- Exposure to someone else's suicidal behavior or suicide death
- Veteran Status and war trauma
- Whether the person is part of a cultural/minority group (e.g., Aboriginal and Torres Strait Island people, LGBTI).

Coping resources and support

- The person's support resources
- Assessment of protective factors e.g. family support, positive coping skills and problem solving skills
- An assessment of family and social connectedness
- Identify the core values/beliefs, goals and strengths of the person.

Ability to recover in the community

- The person's ability to seek and access help - identify any barriers to accessing services including an assessment of financial barriers
- The ability of the person to enter into a therapeutic alliance/partnership including the persons engagement with help
- Identify problem solving strategies the person is open to
- The person's ability to interact with others
- The person's concerns about stigma
- Immediate medium and long term mental health needs
- Immediate and long term social needs.

Role of peer support worker

Offer to:

- advocate for the person to be treated with dignity and respect
- provide basic physical comforts (e.g. blankets, towels, meal trays)
- accompany person while they wait for the SRT/MH team
- help the person communicate with staff in the acute setting
- liaise with the persons family/friends/carer (with the person's consent).

Discharge care plan

Treatment and support recommendations

- Recommendations and actions that address needs identified in the comprehensive psychosocial assessment
- Instructions for medication including frequency, dosage and side effects
- A schedule of appointments for follow-up and aftercare including contact details
- Contingency arrangements for contacting specialists
- Arrangements to overcome barriers to accessing aftercare services
- Housing support recommendations/action needed to secure accommodation (if applicable)
- Recommendations to reduce social isolation and engage with a support network.

Relapse prevention recommendations

- Specific steps to seek help and support if symptoms re-occur or worsen or the situation deteriorates following discharge
- Follow-up procedures following non-compliance or failure to attend aftercare appointments
- Education about warning signs of possible relapse and what to do
- Strategies to mitigate intolerable distress, pain, coping, and suicidal thoughts at home
- Harm minimisation plan for alcohol and drug use (if applicable).

Emergency contact details

- Details of the person's nominated support network and related contact details
- 24/7 mental health emergency contact details (including phone support services such as Lifeline)
- Contact details for crisis assistance and community mental health services.

Discharge summary

- Condition/diagnosis/crisis
- Reason for attending the acute setting
- Summary of assessments performed
- Interventions undertaken by the SRT or MH team
- Summary of the discharge care plan
- Interventions and referrals arranged
- Recommendations to the person GP if the person needs additional help.

Populations requiring assertive follow-up

- With a suspected diagnosed mental illness
- At risk of becoming homeless
- In situation of domestic violence
- Who cannot be sure they can keep themselves safe
- Presenting for suicide risk for the first time
- With a poor history of treatment adherence.

Assertive follow-up includes

- Frequent contact with person, home-visits, intensive case management and out-reach support
- First contact should occur within 24 hours.
